# Tamoxifen Derivatives Alter Retromer-Dependent Endosomal Tubulation and Sorting to Block Retrograde Trafficking of Shiga Toxins

**DOI:** 10.3390/toxins13060424

**Published:** 2021-06-15

**Authors:** Andrey S. Selyunin, Karinel Nieves-Merced, Danyang Li, Stanton F. McHardy, Somshuvra Mukhopadhyay

**Affiliations:** 1 Division of Pharmacology and Toxicology, Institute for Neuroscience, College of Pharmacy, The University of Texas at Austin, Austin, TX 78712, USA; andrey.selyunin@gmail.com (A.S.S.); danyangli@utexas.edu (D.L.); 2 Center for Innovative Drug Discovery, Department of Chemistry, University of Texas San Antonio, San Antonio, TX 78249, USA; karinel.nieves-merced@utsa.edu

**Keywords:** Shiga toxin, Shiga toxin 2, tamoxifen, retromer, trafficking, Golgi, endosome, derivatives, medicinal chemistry

## Abstract

Shiga toxin 1 and 2 (STx1 and STx2) undergo retrograde trafficking to reach the cytosol of cells where they target ribosomes. As retrograde trafficking is essential for disease, inhibiting STx1/STx2 trafficking is therapeutically promising. Recently, we discovered that the chemotherapeutic drug tamoxifen potently inhibits the trafficking of STx1/STx2 at the critical early endosome-to-Golgi step. We further reported that the activity of tamoxifen against STx1/STx2 is independent of its selective estrogen receptor modulator (SERM) property and instead depends on its weakly basic chemical nature, which allows tamoxifen to increase endolysosomal pH and alter the recruitment of retromer to endosomes. The goal of the current work was to obtain a better understanding of the mechanism of action of tamoxifen against the more disease-relevant toxin STx2, and to differentiate between the roles of changes in endolysosomal pH and retromer function. Structure activity relationship (SAR) analyses revealed that a weakly basic amine group was essential for anti-STx2 activity. However, ability to deacidify endolysosomes was not obligatorily necessary because a tamoxifen derivative that did not increase endolysosomal pH exerted reduced, but measurable, activity. Additional assays demonstrated that protective derivatives inhibited the formation of retromer-dependent, Golgi-directed, endosomal tubules, which mediate endosome-to-Golgi transport, and the sorting of STx2 into these tubules. These results identify retromer-mediated endosomal tubulation and sorting to be fundamental processes impacted by tamoxifen; provide an explanation for the inhibitory effect of tamoxifen on STx2; and have important implications for the therapeutic use of tamoxifen, including its development for treating Shiga toxicosis.

## 1. Introduction

Shiga toxin producing *E. coli* (STEC) bacteria are an important cause of food-borne disease that affect over 100,000 individuals in the US and ~3 million people globally each year [1,2,3,4]. Patients initially develop gastrointestinal disease that is usually self-limiting [2,3]. In a subset (~5–15%), Shiga toxin 1 and 2 (STx1 and STx2) produced by the bacteria spread systemically and induce life-threatening or fatal renal disease [2,3]. STx2 is more toxic than STx1 in vivo [5], and disease severity correlates with STx2 production [6]. Currently, antidotes are not available for STx1 or STx2, and antibiotic therapy is contraindicated as it may increase STx1/STx2 release and the risk of renal complications [2,7,8,9,10]. There is also a concern that STx1/STx2 may be converted into biological weapons [11]. In totality, STEC infections are a major global public health problem for which effective interventions are urgently required.

STx1 and STx2 are AB_5_ toxins formed by the association of an enzymatically active A-subunit, which kills host cells by blocking ribosomal protein synthesis, with a B-subunit pentamer, which mediates retrograde trafficking from the cell exterior to the cytosol [12,13,14]. STx1 is essentially identical to STx produced by *Shigella* bacteria (the only difference is a single conservative serine-to-threonine substitution in the A-subunit) while STx2 shares ~55% sequence identity with STx/STx1 [13,14]. The trafficking of STx/STx1 has been extensively characterized. After endocytosis, STx/STx1 sequentially transits through early endosomes, the Golgi apparatus, and the endoplasmic reticulum from where the A-subunit translocates to the cytoplasm [13,14]. Direct transport from early endosomes to the Golgi apparatus allows the toxin to avoid degradation in late endosomes/lysosomes [13,14,15,16,17]. More recent studies from our group revealed that STx2 utilizes the same retrograde pathway as STx1 to access the cytosol and also evades degradation in late endosomes by undergoing direct early endosome-to-Golgi transport [17,18]. The requirement of retrograde trafficking for toxicity of STx/STx1 and STx2 has led to considerable interest in developing inhibitors of toxin trafficking for therapy. Over the last few years, several groups, including ours, have developed experimental transport inhibitors that successfully protect cells and animals against STx/STx1 and/or STx2 toxicity in laboratory settings [13,14,16,19,20,21]. However, none of these inhibitors are approved for use in humans, highlighting the need for additional drug development and pre-clinical studies.

To comprehensively identify druggable host factors required for the trafficking and toxicity of STx2, which is more relevant for human disease [6], we recently carried out a genome-wide siRNA screen [14,21,22]. A major outcome was the discovery that late endosome–lysosome fusion is required for the early endosome-to-Golgi trafficking of STx2 [14,21]. Based on these findings, and the fact that small molecules that deacidify endolysosomes also inhibit the fusion of late endosomes with lysosomes [23,24], we screened for the activity of FDA-approved drugs that alter endolysosomal pH against STx2. Through these studies, we identified tamoxifen, currently used to treat breast cancer [25,26,27], to be a potent inhibitor of the trafficking and toxicity of STx2, as well as STx1, in cells and mice [14,21]. Tamoxifen blocks STx2 transport at the early endosome-to-Golgi step and reroutes the toxin to late endosomes/lysosomes for degradation [14,21]. While tamoxifen is a well-known selective estrogen receptor modulator (SERM) [27], our studies indicated that its activity against STx2 is independent of SERM property [14,21]. Instead, inhibitory activity against STx2 appeared to depend on a relatively understudied lysosomotropic weak base property of tamoxifen [14,21], which was previously demonstrated to confer tamoxifen the ability to accumulate in and directly increase endolysosomal pH [28,29]. We validated the lysosomotropic effect of tamoxifen and further reported that tamoxifen altered the endosomal association of retromer [21], which is required for the early endosome-to-Golgi trafficking of STx1 and STx2 [14,18,30,31,32]. As the recruitment of retromer to endosomes depends on endosomal maturation [33,34,35], which in turn depends on late endosome-lysosome fusion [36], we concluded that a tamoxifen-mediated change in endolysosomal pH likely altered endosomal retromer dynamics to block STx2 trafficking. However, whether endolysosomal deacidification was the primary event that altered retromer dynamics to block toxin trafficking or whether tamoxifen had a pH-independent effect on retromer function had not been definitively answered. Additionally, a clear understanding of the mechanism by which changes in retromer function impacted STx2 trafficking was lacking.

To address the fundamental questions raised above and obtain a better understanding of the mechanism of action of tamoxifen against STx2, herein we performed a combination of structure activity relationship (SAR) studies and mechanistic assays in cell culture. Our results show that the weakly basic nature of tamoxifen provided by an amine group is required, but the ability to change endolysosomal pH is not obligatory. Rather, our studies imply that an inhibition of retromer-dependent endosomal tubulation and sorting is the major mechanism underlying the block in STx2 trafficking. These findings identify a previously unappreciated, non-estrogenic effect of tamoxifen that should aid in the development of tamoxifen compounds as a treatment of STEC infections, and also has important ramifications for the chemotherapeutic use of tamoxifen.

## 2. Results

### 2.1. Structure Activity Relationships

Tamoxifen is a weak base due to the presence of a tertiary amine in its structure. Our previous studies provided preliminary evidence suggesting that the amine group and weakly basic nature were critical for activity against STx2—three clinically approved tamoxifen derivatives with the tertiary amine base, toremifene, raloxifene, and bazedoxifene, were as effective as tamoxifen in inhibiting STx2; however, the derivative ospemifene, which does not contain a basic amine group, lacked activity [14,21]. To definitively determine the role of the amine group and elucidate other structural features required for activity against STx2, we performed a brief set of SAR studies. We treated cells with each generated compound at a final concentration of 10 µM for 24 h, subsequently exposed cells to varying amounts of STx2 in the continuous presence of the compound for an additional 18 h, and then assayed for cell viability. We included vehicle DMSO and tamoxifen as negative and positive controls, respectively, in each assay.

**Figure 1:**
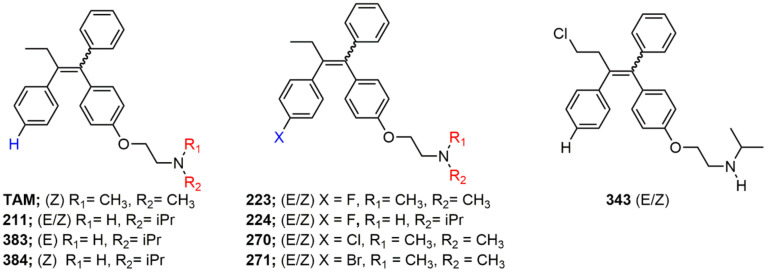
Structures of tamoxifen and derivatives. TAM, tamoxifen.

**Figure 2:**
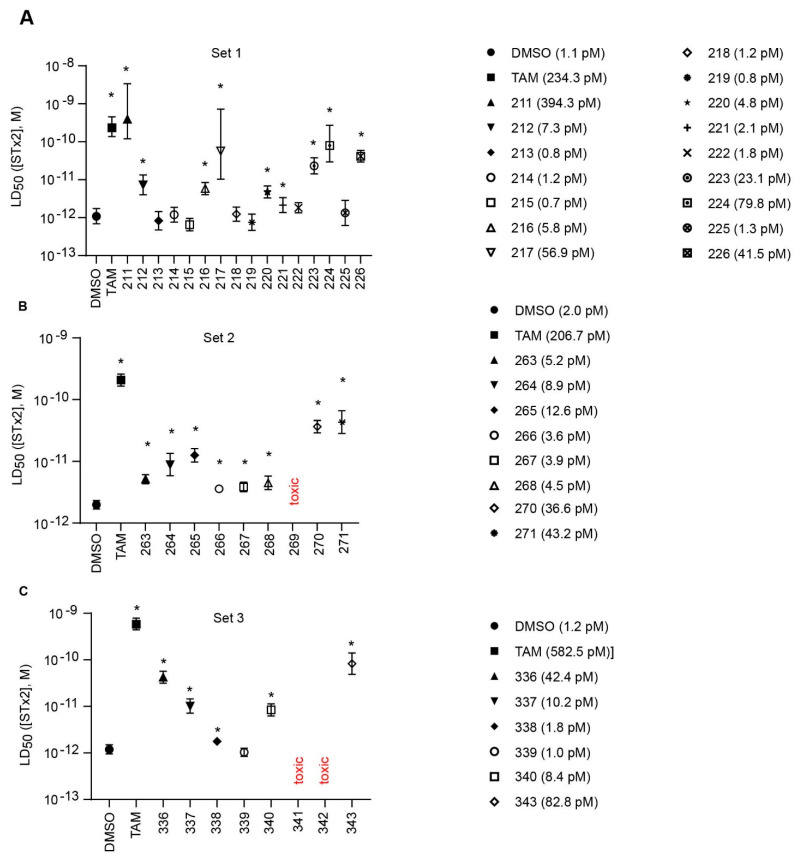
Structure activity relationship studies for tamoxifen derivatives against STx2 toxicity. (**A**–**C**). Cell viability assays in cells treated with DMSO or 10 µM indicated compounds for 24 h prior to the addition of increasing concentrations of STx2 for an additional 18 h. LD_50_ with 95% confidence interval is shown. TAM, tamoxifen. Numbers in parentheses are LD_50_ values. N = 3 per compound. * *p* < 0.05 by non-linear regression for the difference between DMSO and other conditions. Statistical differences between TAM and other conditions using similar analyses are depicted in Table 2.

Due to the abundance of tamoxifen-SERM SAR and synthesis reported in the literature [37,38,39,40,41,42,43,44,45], we focused our SAR studies on the amine (R1 and R2), aryl group substitution (X), and the ethyl group to elucidate which structural features and properties were required for STx2 activity (Figure 1 and Table 1). A total of 36 analogs of tamoxifen were prepared and evaluated in three separate sets of STx2 experiments. All compounds were initially evaluated for STx2 activity as an *E/Z* mixture of double bond isomers. We immediately observed that the dimethyl amine present in tamoxifen could be substituted by a secondary amine (R1 = H, R2 = isopropyl) while still maintaining STx2 activity, as represented by 211 and 224 (Figure 1 and Figure 2A and Table 2; also see 383 and 384 later). Halogenation of the 2-phenyl group at the four position with either F, Cl, or Br (223, 270, and 271) was well tolerated, producing robust STx2 activity (Figure 1 and Figure 2A,B, and Table 2). The ethyl group could also be modified to the 2-chloroethyl moiety, while still producing substantial STx2 activity (343) (Figure 1 and Figure 2C and Table 2). However, the STx2 activity within these analogs was quite specific to either hydrogen or small alkyl groups on the nitrogen, as larger groups such as benzyl, *tert*-butyl, or cyclohexane diminished STx2 activity (214, 340, and 341; Figure 2A,C and Table 2). Connection of the R1 and R2 groups into cyclic structures such as piperidine, piperazines, or related heterocycles also decreased STx2 activity (216, 217, and 218; Figure 2A and Table 2). Holding the dimethyl amine of tamoxifen constant with systematic removal of the 1-phenyl, 2-phenyl, or ethyl group produced analogs with little or no STx2 activity (264, 265, and 266; Figure 2B and Table 2). Finally, saturation of the double bond in tamoxifen completely diminished all STx2 activity (263; Figure 2B and Table 2).

**Figure 3:**
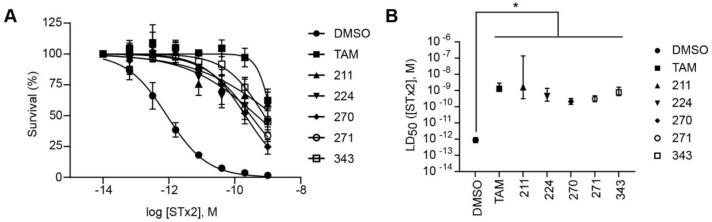
Validation of the protective effect of selected compounds against STx2. (**A**,**B**). Cell viability assays as described in Figure 2. LD_50_ with 95% confidence interval is depicted in (**B**). TAM, tamoxifen. N = 3 per compound. * *p* < 0.05 by non-linear regression for the difference between DMSO and other conditions.

We selected the top two protective derivatives from the first two sets and top protector from the third set for validation in an independent experiment (i.e., compounds 211 and 224 from Figure 2A; 270 and 271 from Figure 2B; and 343 from Figure 2C; also see Table 2). Only one compound was used from the third set because the extent of protection provided by the other compounds in this set was substantially lesser than that of tamoxifen and the other selected compounds (see comparison of fold difference between STx2 LD_50_ of tamoxifen and derivatives in Table 2). Among the compounds selected for further processing, 211 and 224 were as protective as tamoxifen, while the other compounds provided lesser protection (Figure 2A–C; Table 2). The new experiment validated the protective effects of all selected compounds (Figure 3A,B). Subsequent studies focused on compound 211 because it was the only compound that provided a higher LD_50_ of STx2 than tamoxifen (Figure 2A and Figure 3A,B and Table 2; note, however, that there was no statistical difference between the LD_50_ of STx2 in 211 or tamoxifen-treated cells).

### 2.2. The Protective Function of Compound 211 Is Stereospecific

**Figure 4:**
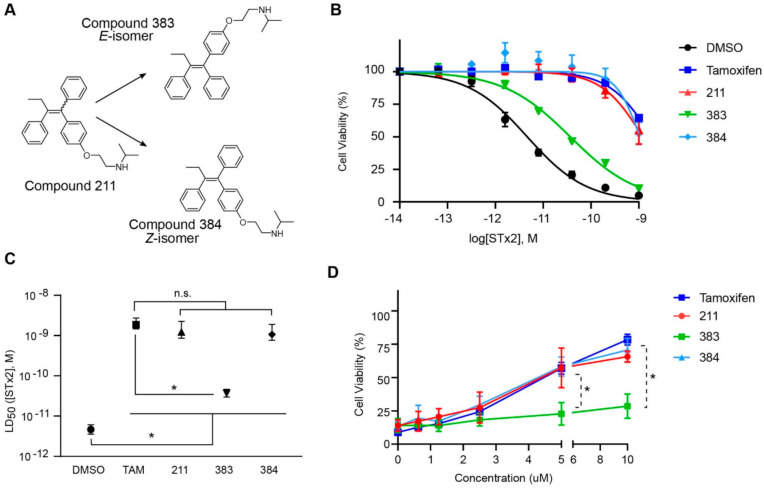
Stereoisomers of compound 211 show variable protective effect against STx2 toxicity. (**A**). Chemical structures of *E-* and *Z-* isomers of compound 211. (**B**,**C**). Cell viability assays after pre-treatment with DMSO or 10 µM indicated compounds for 24 h followed by exposure to STx2 for 18 h. LD_50_ with 95% confidence interval is depicted in (**C**). TAM, tamoxifen. N = 3 per compound. * *p* < 0.05 by non-linear regression for indicated differences; n.s., not significant. (**D**). Cell viability using varying concentrations of indicated compounds and 40 pM STx2. Other conditions were identical to (**B**) above. TAM, tamoxifen. N = 3 for each treatment. * *p* < 0.05 by two-way ANOVA with drug treatment and concentration as independent variables and Tukey–Kramer post hoc test for indicated comparisons.

Compound 211 and related stereoisomers have the opportunity to exist as geometric isomers at the tetra-substituted alkene moiety, allowing for either *E-* or *Z-*configurations (Figure 4A). The active pharmaceutical ingredient (API) for tamoxifen is the *Z-*isomer, which has a 100-fold higher affinity for the estrogen receptor than the corresponding *E-*isomer. While the effect of tamoxifen on STx2 transport is independent of estrogen signaling [14,21], determining whether the protective effect of compound 211 was related to stereochemistry was expected to provide important insights about the underlying molecular mechanisms. Notably, the *E-*isomer (compound 383) exhibited only mild protective activity against STx2, whereas the *Z-*isomer (compound 384) was comparable to the native racemic preparation (compound 211) and tamoxifen (Figure 4B,C). We confirmed these results using a dose response experiment in which cells were exposed to varying levels of each compound and a fixed amount of STx2. Tamoxifen, compound 211, and 384 were protective when used at a concentration of 5 µM or higher (Figure 4D), and at these concentrations, the protective effects of tamoxifen, 211, and 384 were comparable to each other and substantially higher than 383 (Figure 4D).

### 2.3. Protective Compounds Block Retrograde Trafficking of STx2

**Figure 5:**
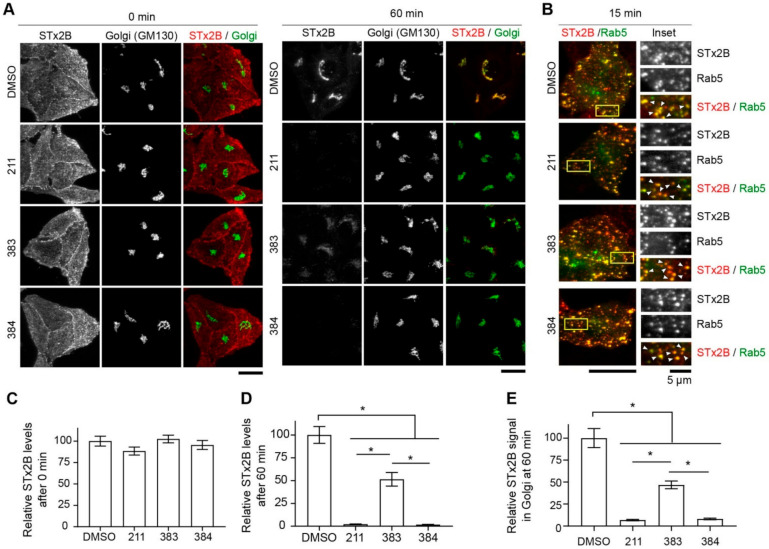
Compounds 383 and 384 inhibit transport of STx2B to the Golgi. (**A**). STx2B transport assay in cells treated with DMSO (0.1%) or 10 µM indicated compounds for 24 h. Cells were fixed at 0 or 60 min after the start of transport and processed for microscopy. Scale bars, 25 µm. (**B**). STx2B transport assay in cells transfected with GFP-Rab5 for 4 h followed by treatment with DMSO (0.1%) or 10 µM indicated compounds for 24 h. Cells were fixed 15 min after the start of transport. Scale bar, 25 µm; inset, 5 µm. Arrows, overlap. (**C**,**D**). Quantification of STx2B signal from (**A**). DMSO-treated cells at 0 min normalized to 100. N ≥ 25 cells per condition. * *p* < 0.05 by one-way ANOVA and Tukey–Kramer post hoc test for indicated comparisons. (**E**). Quantification of Golgi-localized STx2B signal from (**A**). Signal intensity after DMSO treatment at 60 min is normalized to 100. N ≥ 25 cells per condition. * *p* < 0.05 by one-way ANOVA and Tukey–Kramer post hoc test for indicated comparisons.

**Figure 6:**
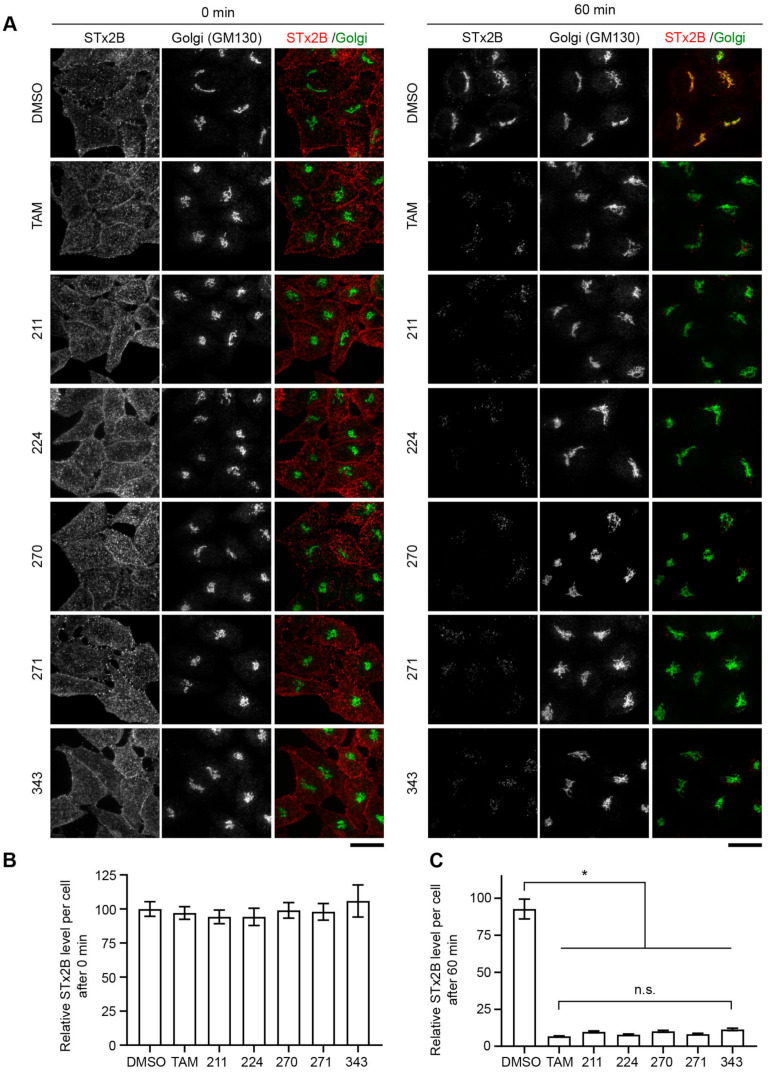
Compounds 211, 224, 270, 271, and 343 are as effective as tamoxifen (TAM) in inhibiting retrograde trafficking of STx2B. (**A**). STx2B transport assay after exposure to DMSO (0.1%) or 10 µM indicated compounds for 24 h. Cells were fixed at 0 or 60 min after the start of transport. Scale bars, 25 µm. (**B**,**C**). Quantification of STx2B signal from (**A**). DMSO-treated cells at 0 min normalized to 100. N ≥ 25 cells per condition. * *p* < 0.05 by one-way ANOVA and Tukey–Kramer post hoc test for indicated comparisons; n.s., not significant.

Next, we tested whether the protective effects of compounds 211, 383, and 384 were related to their ability to inhibit STx2 trafficking. As expected from our earlier work [18,21,22], in vehicle DMSO treated cells, the B-subunit of STx2 (STx2B) bound the cell surface at the start of the transport assay, trafficked to Rab5-positive early endosomes at 15 min, and reached the Golgi apparatus at 60 min (Figure 5A–E). Similar to our prior results with tamoxifen [21], which are also reproduced here in Figure 6 later, after treatment with 211 or 384, STx2B trafficked to early endosomes, but then failed to transit to the Golgi apparatus and instead was degraded (Figure 5A–E). Notably, while compound 383 also inhibited trafficking of STx2B to the Golgi apparatus and led to the degradation of STx2B, it was not as efficient as 211 or 384 (Figure 5A–E). Indeed, at the 60 min time-point, in compound 383-treated cells, total cellular and Golgi STx2B levels were lesser than DMSO vehicle, but greater than the other compounds (Figure 5A–E). In a separate experiment, we verified that compound 211 was as effective as tamoxifen in inhibiting STx2B trafficking and inducing STx2B degradation (Figure 6A–C; this experiment also demonstrated that compounds 224, 270, 271, and 343 robustly inhibited STx2B trafficking). In totality, data in Figure 3, Figure 4, Figure 5 and Figure 6 indicate that compounds 211, 383, and 384 protect against STx2 toxicity by blocking toxin trafficking. Additionally, the results of the trafficking assays are consistent with the cell viability experiments and imply that the *Z*-isomer of compound 211 is the active compound.

### 2.4. Relationship between Deacidification of Endolysosomes and Inhibition of STx2 Trafficking

**Figure 7:**
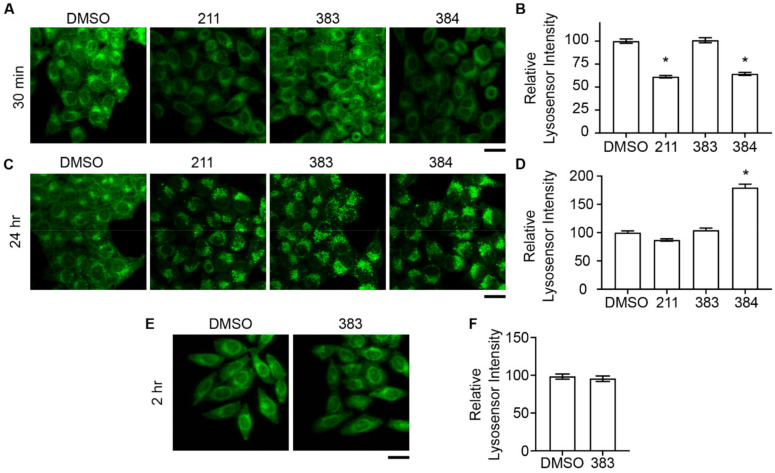
Variable effect of compounds 211, 383, and 384 on lysosomal acidification. (**A**,**C**,**E**). LysoSensor staining in cells treated with DMSO or 10 µM indicated compounds for 30 min (**A**), 24 h (**C**), or 2 h (**E**). (**B**,**D**,**F**). Quantification of mean LysoSensor signal per cell from (**A**,**C**,**E**). Signal in DMSO-treated cells is normalized to 100. N ≥ 100 cells per condition. * *p* < 0.05 by one-way ANOVA and Tukey–Kramer post hoc test for comparison between DMSO and other treatments (**B**,**D**). Scale bars, 25 µm.

Several lines of evidence from our previous work suggested that tamoxifen compounds may inhibit retrograde trafficking of STx2B by deacidifying endolysosomes [14,21]. To summarize here: (1) tamoxifen was known to rapidly increase the pH of endolysosomes [28,29,46], and we verified this result [21]; (2) tamoxifen derivatives that protected against STx2 contained the tertiary amine base necessary for weak base activity and endolysosomal deacidification [21]; and (3) deacidification of endolysosomes impacts fusion of late endosomes with lysosomes [23,24], which we reported is required for the early endosome-to-Golgi transport of STx2B [14,21]. Notably, our studies showed that tamoxifen increased endolysosomal pH soon after treatment (30 min), but inhibition of STx2B transport required longer exposure (24 h) by which time endolysosomal pH had returned to normal (although morphological changes in the endolysosomal compartments remained) [21]. The results about the transient effect on endolysosomal pH were consistent with a previous report demonstrating that tamoxifen increased endolysosomal pH 30 min after exposure, but restoration towards baseline occurred soon thereafter at 60 min [46]. We interpreted these findings to mean that an initial change in endolysosomal pH initiated a sequence of events that subsequently led to a block in STx2 trafficking [21]. Based on the above, we expected that compound 383, which had a reduced inhibitory effect on STx2 trafficking, would also have a reduced capability to deacidify endolysosomes. We observed that compounds 211 or 384 increased endolysosomal pH within 30 min, which returned to or below baseline by 24 h (Figure 7A–D). Surprisingly, however, compound 383 did not have any effect on endolysosomal pH at the 30 min or 24 h time-point (Figure 7A–D). Since tamoxifen and related lysosomotropic agents have a rapid and transient effect on endolysosomal pH, the likelihood that 383 had a delayed effect on endolysosomal acidity was low. Nevertheless, to rule out this possibility, we repeated the assay at an intermediate time-point (2 h) and did not observe any effects (Figure 7E,F). Overall, our results indicate that compound 383 does not deacidify endolysosomes. As 383 exerts reduced, but measurable, inhibitory activity against STx2 trafficking and toxicity, these results imply that deacidification of endolysosomes may contribute to, but is not obligatorily required for, the anti-STx2 activity of tamoxifen compounds.

### 2.5. Tamoxifen Compounds Alter Retromer-Mediated Endosomal Tubulation and Sorting

**Figure 8:**
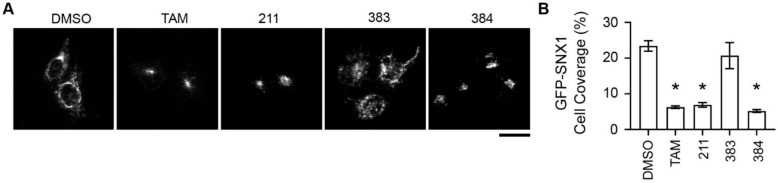
Tamoxifen compounds alter the cellular distribution of GFP-SNX1 endosomes. (**A**). Microscopy assays in cells transfected with GFP-SNX1 for 4 h and treated with indicated compounds for an additional 24 h. Scale bar, 25 µm. (**B**). Quantification of cell surface coverage of the GFP-SNX1 signal from (**A**). TAM, tamoxifen. N = 25 cells per treatment. * *p* < 0.05 by one-way ANOVA and Tukey–Kramer post hoc test for comparison between DMSO and other conditions.

**Figure 9:**
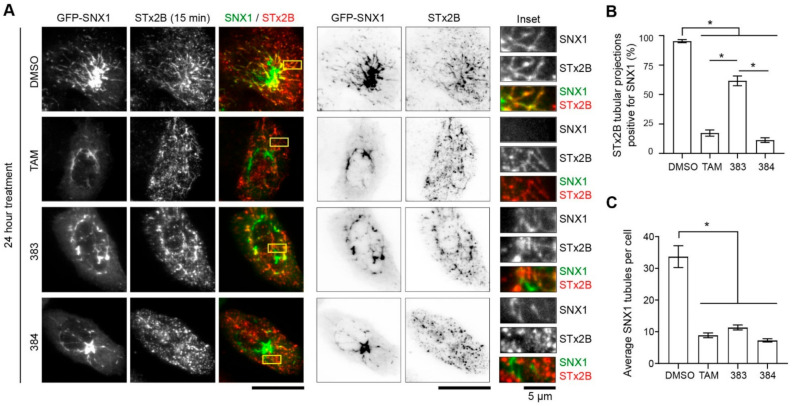
Tamoxifen compounds interfere with SNX1-dependent endosomal tubulation and sorting. (**A**). STx2B transport assay in cells transfected with GFP-SNX1 for 4 h followed by treatment with 10 µM indicated compounds for 24 h. Cells were fixed 15 min after the start of transport. Scale bars, 25 µm; inset, 5 µm. (**B**). Quantification of STx2B tubular extensions overlapping with SNX1 signal from (**A**). N = 15 cells per treatment. * *p* < 0.05 by one-way ANOVA and Tukey–Kramer post hoc test for indicated comparisons. (**C**). Quantification of SNX1 tubulation from (**A**). N ≥ 14 cells per treatment. * *p* < 0.05 by one-way ANOVA and Tukey–Kramer post hoc test for indicated differences.

Retromer is a coat protein that cycles on-and-off endosomal membranes, produces Golgi-directed membrane tubules from endosomes, and mediates early endosome-to-Golgi transport of many cargo proteins, including STx1 and STx2 [13,18,33,34,35]. Our previous studies revealed that tamoxifen treatment altered the recruitment of retromer to early endosomal membranes, and suggested that changes in endosomal retromer dynamics may underlie the observed block in the early endosome-to-Golgi trafficking of STx1/STx2 [21]. We interpreted the effect of tamoxifen on retromer to be a consequence of a tamoxifen-induced deacidification of endolysosomes because (1) retromer recruitment depends on endosomal maturation [33,34,35]; (2) inhibition of late-endosome lysosome fusion impacts endosomal maturation [36]; and (3) as described earlier, deacidification of endolysosomes inhibits late-endosome lysosome fusion [23,24]. However, the fact that compound 383 inhibited STx2B transport in the current study without deacidifying endolysosomes raised doubts about the above interpretation. An alternative possibility is that tamoxifen can partition into lipid membranes due to its lipophilic nature, and partitioning of tamoxifen into endosomes may impact retromer function. A testable hypothesis emerged from this idea—if the effect of tamoxifen compounds on retromer is critical for inhibition of STx2 trafficking, and if this effect is independent of deacidification of endolysosomes, compared with compounds 211 and 384, compound 383 should have a reduced, but measurable, effect on retromer dynamics. To test this hypothesis, we expressed a GFP-tagged construct coding for the retromer component SNX1 and assayed for (1) the cellular distribution of SNX1; (2) total number of SNX1 endosomal tubules; and (3) overlap of SNX1 endosomal tubules with STx2B. We used quantitative changes in SNX1 endosomal tubules as an important experimental end-point because these tubules are the transport intermediates that traffic cargo proteins from endosomes to the Golgi [47]. Further, we utilized GFP-SNX1 transfection instead of staining for endogenous SNX1 because retrograde endosomal tubules are more easily visualized after overexpression of SNX1. For the cellular distribution assay, we observed that, under vehicle treatment conditions, GFP-SNX1 was distributed in the cell periphery with numerous tubular structures visible (Figure 8A,B). Notably, treatment with tamoxifen, 211 or 384, but not 383, led to a collapse of the SNX1 signal to the perinuclear region (Figure 8A,B). This observation is similar to our previous results with endogenous SNX1 [21]. We then assayed for the trafficking of STx2B in cells expressing GFP-SNX1 after treatment with vehicle or various tamoxifen compounds. For this experiment, cells were fixed 15 min after the initiation of transport, which corresponds to the time-point at which STx2B is expected to undergo early endosome-to-Golgi transport. In DMSO-treated cells, we detected numerous SNX1 and STx2B tubules in each cell, and ~90% of STx2B tubules were positive for SNX1 (Figure 9A,B). Treatment with tamoxifen, 383 or 384 strongly inhibited the total number of SNX1 tubules present per cell (Figure 9A–C). Importantly, tamoxifen or 384 treatment robustly reduced the overlap between STx2B and GFP-SNX1 tubules (<25% of STx2B tubules were positive for GFP-SNX1; Figure 9A,B). However, the effect of 383 was more modest and >50% STx2B tubules were positive for GFP-SNX1 (Figure 9A,B). Thus, protective tamoxifen compounds change the cellular distribution of SNX1, reduce formation of SNX1 endosomal tubules, and decrease the sorting of STx2B into SNX1-positive tubules. While compound 383 is as efficient as tamoxifen and 384 in reducing SNX1 endosomal tubules, it has only a partial effect on the sorting of STx2B into SNX1 tubules. Combined with the lack of effect of compound 383 on endolysosomal pH, these results suggest that a primary effect on retromer-mediated endosomal tubulation and sorting is the likely mechanism by which tamoxifen compounds block retrograde trafficking of STx2 to protect against STx2 toxicity.

## 3. Discussion

The identification of tamoxifen as an inhibitor of STx1/STx2 trafficking emerged from our discovery that the early endosome-to-Golgi trafficking of STx2B is dependent on late endosome-lysosome fusion [14,21]; the known inhibitory effect of drugs that increase endolysosomal pH on late endosome-lysosome fusion [23,24]; and the weak base, lysosomotrophic property of tamoxifen [28,29,46]. The tertiary amine group of tamoxifen makes it a weak base. The strong inhibitory activity of tamoxifen derivatives with the tertiary amine base (bazedoxifene, raloxifene, and toremifene) compared with the reduced or lack of activity of compounds that, respectively, are a weaker base due to the presence of a secondary amine (endoxifen) or lack basic activity altogether due to the absence of an amine group (ospemifene) supported the idea that a weak base-mediated lysosomotrophic effect of tamoxifen is responsible for activity against STx2 [14,21]. In contrast with our prior interpretations, an important outcome of the current work is that while the tertiary amine group of tamoxifen is, indeed, necessary to inhibit STx2, an increase in endolysosomal pH is not obligatorily required. This conclusion comes out of the finding that compound 383, which had the tertiary amine base, did not deacidify endolysosomes, but still exhibited measurable inhibitory activity against STx2 trafficking and toxicity. Thus, the anti-STx2 activity of tamoxifen compounds can be separated from their lysosomotropic effects. Note, however, that our current data do not eliminate the possibility that, for tamoxifen compounds with a lysosomotropic effect, deacidification of endolysosomes contributes to the inhibitory activity against STx2.

We had previously reported an effect of tamoxifen on endosomal retromer recruitment [21]. Our current findings extend and modify these observations. We observed that protective tamoxifen compounds reduced the number of endosomal SNX1 tubules and inhibited the sorting of STx2 into SNX1 tubules. Notably, compound 383, which had a modest inhibitory effect on STx2, robustly blocked SNX1 tubulation but had a more limited effect on the sorting of STx2B into SNX1 tubules. Thus, tamoxifen compounds appear to have distinct inhibitory effects on retromer-dependent endosomal tubulation and sorting. The inhibitory effect on sorting, but not tubulation, appears to be predictive of efficacy in blocking retrograde transport. As described earlier, since compound 383 did not deacidify endolysosomes, an implication is that tamoxifen compounds likely have a pH-independent effect on retromer dynamics. Determining the mechanism by which tamoxifen impacts retromer function is now important. A possibility is that the observed effects may be a consequence of the incorporation of tamoxifen into endosomal lipids, which impacts the recruitment and function of retromer independent of other proteins. In vitro liposome tubulation assays should be able to address this hypothesis in the future.

Our previous work revealed that the protective effect of tamoxifen against STx2 is independent of SERM activity. This conclusion emerged from the facts that: (1) HeLa cells, which we used in our assays, do not express estrogen receptors [48]; and (2) ospemifene is a SERM compound [49], but has no activity against STx2 [14,21]. We did not assay for SERM activity of the derivatives generated in this study. However, since our experiments were performed in HeLa cells, an inherent implication is that the protective effects of the derivatives against STx2 are independent of any effects on estrogen signaling.

Our prior study revealed a protective effect of tamoxifen against mice exposed to STx1 or STx2 [21]. It will be critical to determine whether, at the animal level, (1) the protective effect is mediated by the effects of tamoxifen on retromer; and (2) certain dosing or delivery techniques optimize the effects of tamoxifen on retromer while simultaneously reducing SERM or other off-target effects. Delineating the effects of tamoxifen on retromer function and endosome-to-Golgi trafficking in vivo should directly aid efforts to repurpose tamoxifen for the management of STEC infections. Additionally, such studies may also provide important information relevant to the chemotherapeutic use of tamoxifen as some of the observed clinical effects of tamoxifen may be secondary to changes in membrane trafficking rather than modulation of estrogen signaling. Overall, findings described here have important ramifications for the use of tamoxifen in humans.

In conclusion, we show that tamoxifen compounds block STx2 trafficking by inhibiting retromer-dependent endosomal tubulation and sorting, providing a mechanism that may be leveraged for the treatment of Shiga toxicosis.

## 4. Materials and Methods

### 4.1. Generation of Novel Tamoxifen Derivatives

Detailed procedures utilized for compound generation is provided as Supplementary Materials. The structure of each compound is depicted in Table 1.

### 4.2. Plasmids and Reagents

We have previously described the GFP-SNX1 and GFP-Rab5 plasmids used in this study [16]. Monoclonal anti-GM130 (#610822) was from BD Biosciences (San Jose, CA, USA). Tamoxifen (TAM) was purchased from Sigma-Aldrich (St. Louis, MO, USA). Derivative compounds were synthesized at the Center for Innovative Drug Discovery (University of Texas at San Antonio, San Antonio, TX, USA; see Supplementary Materials). All compounds were dissolved in DMSO and used at 10 µM final concentration unless specified otherwise.

STx2 holotoxin and STx2B were obtained from BEI Resources (Manassas, VA, USA). For transport assays, STx2B was labeled using Alexa Fluor™ 555 Microscale Protein Labeling Kit using manufacturer’s instructions (ThermoFischer, Waltham, MA, USA).

### 4.3. Cell Culture and Transfections

A HeLa cell line that stably expresses globotriaosylceramide was used in this study, and we have described this line extensively previously [18,21,22]. Cells were cultured at 37 °C under 5% CO_2_ in Dulbecco’s Modified Eagle Medium/Ham F12 (50/50) (Mediatech, Inc., Manassas, VA, USA) supplemented with 10% fetal bovine serum (Atlanta Biologicals, Flowery Branch, G, USA), 100 IU/mL penicillin-G, and 100 µg/mL streptomycin (both Mediatech).

DNA transfections were performed using JetPEI (VWR International, Randor, PA, USA) following manufacturer’s instructions. In experiments with derivative compounds, cells were transfected for 4 h, and then transferred into media containing indicated compounds for an additional 24 h, unless specified otherwise.

Lysosomal pH was evaluated using LysoSensor Green DND-189 probe (ThermoFisher, Waltham, MA, USA) used at 1 µM. Cells were exposed to the probe in presence of the indicated compounds for 30 min and imaged live immediately. For experiments performed at the 30 min time-point after drug exposure, both LysoSensor and derivatives were added simultaneously. For assays at the 2 or 24 h time-point, LysoSensor was added for the last 30 min of the experiment.

### 4.4. Cytotoxicity Assay

Cells were treated with DMSO (0.1%) or indicated compounds for 24 h, followed by incubation with the indicated concentration of STx2 holotoxin and the compound for an additional 18 h. Cell viability was assayed using (3-(4,5-Dimethylthiazol-2-yl)-2,5-Diphenyltetrazolium Bromide 3-4-5 (MTT) reagent as described by us previously [21,22].

### 4.5. STx2B Transport Assay

Transport of fluorescently labeled STx2B was performed essentially as described previously [18,21,22]. Briefly, cells were washed with ice-cold phosphate buffered saline and incubated with 1 µg/mL of STx2B in ice-cold culture medium for 30 min on ice at 4°C to allow toxin binding to the cell surface. Cells were then washed with ice-cold phosphate buffered saline and transferred to fresh pre-warmed culture medium at 37 °C to initiate toxin transport. Cultures were fixed after start of transport at times indicated in each figure and processed for microscopy. Drugs remained in the media during the transport assay.

### 4.6. Immunofluorescence Microscopy, Imaging, Quantitative Analysis, and Statistics

We used protocols described previously by us for performing immunofluorescence staining [18,21,22]. For imaging, a swept-field confocal microscope equipped with a four-line high-power laser launch (Nikon) and an iXon3 X3 DU897 electron-multiplying charge-coupled device camera (Andor Technology) was used. For all experiments except live-imaging using LysoSensor, a 100×, 1.45 numerical aperture oil objective was used. Live imaging assays utilized a 60×, 1.4 numerical aperture oil immersion objective. Additionally, for live-cell assays, imaging was in 35-mm glass bottom plates using a water-jacketed live imaging stage set to 42 °C in DMEM/F12 medium supplemented with 10% fetal bovine serum and penicillin/streptomycin. Live imaging was performed within 10 min of removing from the incubator. All images were captured as z-stacks. Images depicted in the figures are maximum-intensity projections of the stacks.

All computational analyses were performed using ImageJ (National Institutes of Health; http://rsb.info.nih.gov/ij/index.html). Average fluorescence levels per cell and within the Golgi were calculated using average projections as described previously [18].

To calculate the relative cell coverage of GFP-SNX1 tubules, cells were outlined using a free hand selection tool and the area was measured using ‘Set Measurements—Area’ function. The SNX1 signal was isolated to the boundary of the cell outlined prior and processed as a binary via the threshold function using identical parameters across all treatments. The area of the SNX1 binary signal was measured as above using ‘Set Measurements—Area’ function. The ratio of the SNX1 signal area to the cell outline area was then calculated and graphed.

GFP-SNX1 tubules per cell were calculated by manually outlining non-punctate extensions in individual cells and quantifying the number of elements with length > 0.5 µm.

Quantification of STx2B in SNX1 tubules was performed by selecting twenty 1 µm × 1 µm ROIs over non-punctate STx2B signal in each cell and assessing for clear SNX1 signal in corresponding ROI. Areas with overlapping STx2B and SNX1 signal were scored as positive.

Statistical analyses were performed using GraphPad Prism 8 software (GraphPad, San Diego, CA, USA). All cell culture experiments were independently replicated at least three times. For microscopy, cells required to attain the sample size provided in individual figure legends came from multiple images captured under identical imaging conditions. For comparisons between multiple groups, one- or two-way ANOVA followed by Dunnett’s or Tukey–Kramer post hoc test was used. Nonlinear regression was used to calculate and compare the LD_50_ of STx2. In all analyses, *p* < 0.05 was considered statistically significant. Asterisks in graphs represent statistically significant differences.

## Figures and Tables

**Table 1:** Tamoxifen analogs in SAR studies.

Molecule Name	Structure	Lot ID	Molecule Name	Structure	Lot ID
CIDD-0000574 ^1^	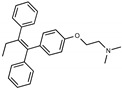	Prestw-146 905 466 SM2019-96-70b1	CIDD-0150264	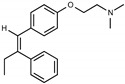	SM2019-96-107b
CIDD-0150211	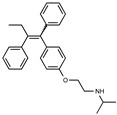	SM2019-96-67b SM2019-96-150c SM2019-96-172c1	CIDD-0150265	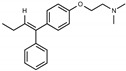	SM2019-96-111b
CIDD-0150212	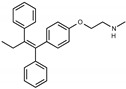	SM2019-96-69b	CIDD-0150266	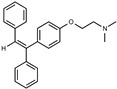	SM2019-96-113b
CIDD-0150213	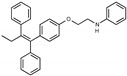	SM2019-96-71a	CIDD-0150267	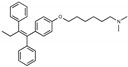	SM2019-96-120b
CIDD-0150214	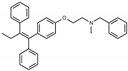	SM2019-96-72b	CIDD-0150268	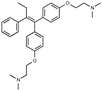	SM2019-96-102-2b
CIDD-0150215	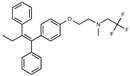	SM2019-96-73b	CIDD-0150269	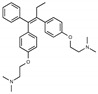	SM2019-96-134b
CIDD-0150216	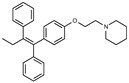	SM2019-96-74b	CIDD-0150270	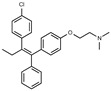	SM2019-96-132b
CIDD-0150217	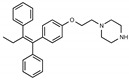	SM2019-96-75b2	CIDD-0150271	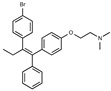	SM2019-96-133b
CIDD-0150218	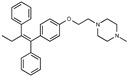	SM2019-96-76b1	CIDD-0150336	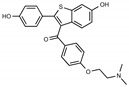	SM2019-96-138b
CIDD-0150219	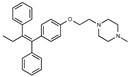	SM2019-96-78a2	CIDD-0150337	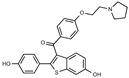	SM2019-96-139b
CIDD-0150220	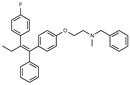	SM2019-96-79a	CIDD-0150338	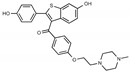	SM2019-96-140b
CIDD-0150221	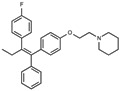	SM2019-96-80b	CIDD-0150339	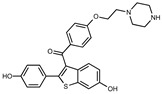	SM2019-96-147b
CIDD-0150222	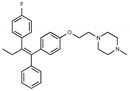	SM2019-96-81a	CIDD-0150340	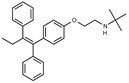	SM2019-96-143b
CIDD-0150223	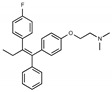	SM2019-96-83c	CIDD-0150341	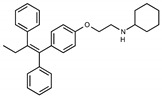	SM2019-96-144b
CIDD-0150224	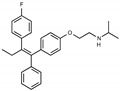	SM2019-96-84b	CIDD-0150342	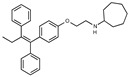	SM2019-96-145b
CIDD-0150225	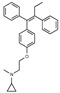	SM2019-96-85b	CIDD-0150343	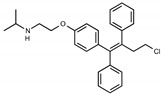	SM2019-96-148b1
CIDD-0150226	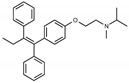	SM2019-96-86b	CIDD-0150383	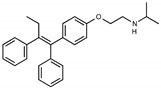	SM2019-96-150c1
CIDD-0150263	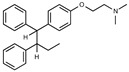	SM2019-96-92	CIDD-0150384	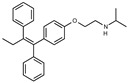	SM2019-96-150c4

^1^ For simplicity, the last three digits are used to identify all molecules throughout the article.

**Table 2:** STx2 LD_50_ values with 95% confidence interval (CI) from Figure 2. Y or N indicates “yes” or “no” to the question whether LD_50_ values are statistically different from DMSO or tamoxifen (TAM) (*p* < 0.05) using non-linear regression.

Compound	LD_50_ (95% CI) (pM)	Fold of DMSO (Different?)	Fold of TAM (Different?)
**Figure 2A**			
DMSO	1.1 (0.7–1.7)	-	0.005 (Y)
TAM	234.3 (136.0–454.4)	213 (Y)	-
211	394.3 (120.0–3407.0)	358 (Y)	1.68 (N)
212	7.3 (4.0–13.4)	6.64 (Y)	0.031 (Y)
213	0.8 (0.5–1.5)	0.73 (N)	0.003 (Y)
214	1.2 (0.8–1.9)	1.09 (N)	0.005 (Y)
215	0.7 (0.4–1.0)	0.64 (N)	0.003 (Y)
216	5.8 (4.0–8.5)	5.27 (Y)	0.025 (Y)
217	56.9 (10.4–723.0)	51.7 (Y)	0.243 (N)
218	1.2 (0.8–1.9)	1.09 (N)	0.005 (Y)
219	0.8 (0.5–1.2)	0.73 (N)	0.003 (Y)
220	4.8 (3.3–6.8)	4.36 (Y)	0.020 (Y)
221	2.1 (1.4–3.4)	1.91 (Y)	0.009 (Y)
222	1.8 (1.3–2.5)	1.64 (N)	0.008 (Y)
223	23.1 (14.2–37.8)	21.0 (Y)	0.099 (Y)
224	79.8 (29.4–272.7)	72.5 (Y)	0.341 (N)
225	1.3 (0.6–2.9)	1.18 (N)	0.006 (Y)
226	41.5 (29.3–59.2)	37.7 (Y)	0.177 (Y)
**Figure 2B**			
DMSO	2.0 (1.7–2.3)	-	0.010 (Y)
TAM	206.7 (165.3–259.4)	103 (Y)	-
263	5.2 (4.4–6.1)	2.60 (Y)	0.025 (Y)
264	8.9 (5.9–13.6)	4.45 (Y)	0.043 (Y)
265	12.6 (9.8–16.2)	6.30 (Y)	0.061 (Y)
266	3.6 (3.2–4.1)	1.80 (Y)	0.017 (Y)
267	3.9 (3.2–4.6)	1.95 (Y)	0.019 (Y)
268	4.5 (3.5–5.8)	2.25 (Y)	0.022 (Y)
269	toxic	-	-
270	36.6 (29.0–46.2)	18.3 (Y)	0.177 (Y)
271	43.2 (28.4–66.0)	21.6 (Y)	0.209 (Y)
**Figure 2C**			
DMSO	1.2 (1.0–1.5)	-	0.002 (Y)
TAM	582.5 (440.7–791.5)	485 (Y)	-
336	42.4 (31.6–57.0)	35.3 (Y)	0.073 (Y)
337	10.2 (7.1–14.5)	8.50 (Y)	0.018 (Y)
338	1.8 (1.6–2.0)	1.50 (Y)	0.003 (Y)
339	1.0 (0.8–1.3)	0.83 (N)	0.002 (Y)
340	8.4 (6.3–11.4)	7.00 (Y)	0.014 (Y)
341	toxic	-	-
342	toxic	-	-
343	82.8 (48.7–141.2)	69.0 (Y)	0.142 (Y)

## Data Availability

The data presented in this study are available within this article and its Supplementary Materials.

## Supplementary Materials

The following are available online at https://www.mdpi.com/article/10.3390/toxins13060424/s1. General chemistry procedures, Experimental procedures for synthesis of tamoxifen derivatives, Figure S1: Synthesis of compounds 211–226 and 340–342.
