# Supplementary material for: Tamoxifen Derivatives Alter Retromer-Dependent Endosomal Tubulation and Sorting to Block Retrograde Trafficking of Shiga Toxins
Source: Toxins (Basel). 2021 Jun 15;13(6):424. doi: 10.3390/toxins13060424 (PMC8232625; doi:10.3390/toxins13060424)
Supplement: Supplementary file 1 [file toxins-13-00424-s001.zip › toxins-1126214-supplementary.pdf]

Supplementary Materials: Tamoxifen Derivatives Alter Retromer-Dependent Endosomal Tubulation and Sorting to Block Retrograde Trafficking of Shiga Toxins

Andrey S. Selyunin, Karinel Nieves-Merced, Danyang Li, Stanton F. McHardy and Somshuvra Mukhopadhyay

General chemistry procedures

Chemicals were purchased from established commercial suppliers and used without further purification. Solvents used for reactions were indicated as of commercial dry or extra-dry or analytical grade. Unless otherwise indicated all reactions were conducted in standard commercially available glassware using standard synthetic chemistry methods and setup. All air- and moisture-sensitive reactions were performed under nitrogen atmosphere with dried solvents and glassware under anhydrous conditions. Analytical thin-layer chromatography (TLC) was carried out using silica gel 60 F_{254} TLC plates. TLC visualization was achieved with a UV lamp or by staining in an iodine chamber. Flash column chromatography was done on a system using prepacked silica gel columns or using silica gel 60A (230–400 mesh) or with preparative thin-layer chromatography plates (1000 micron F_{254}), or using a Biotage Isolera One 2.2, using commercial columns that were pre-packed with Merck Kieselgel 60 (230–400 mesh) silica gel. NMR spectra were recorded on Agilent DD2 400MHz and Bruker 500 MHz Avance III HD spectrometers at ambient temperature. Samples were dissolved and prepared in deuterated solvents ($CDCl_3$, CD_3OD, and DMSO-d_6) with residual solvents being used as the internal standard in all cases. Chemical shifts (δ) are given in parts per million (ppm) and coupling constants (*J*) are given in Hertz (Hz). The proton spectra are reported as follows: d (multiplicity, coupling constant *J*, number of protons). The following abbreviations were used to explain the multiplicities: s = singlet, d = doublet, t = triplet, q = quartet, p = pentet, sext = sextet, sep = septet, and m = multiplet.

Synthesis of Compounds 211–226 and 340–342

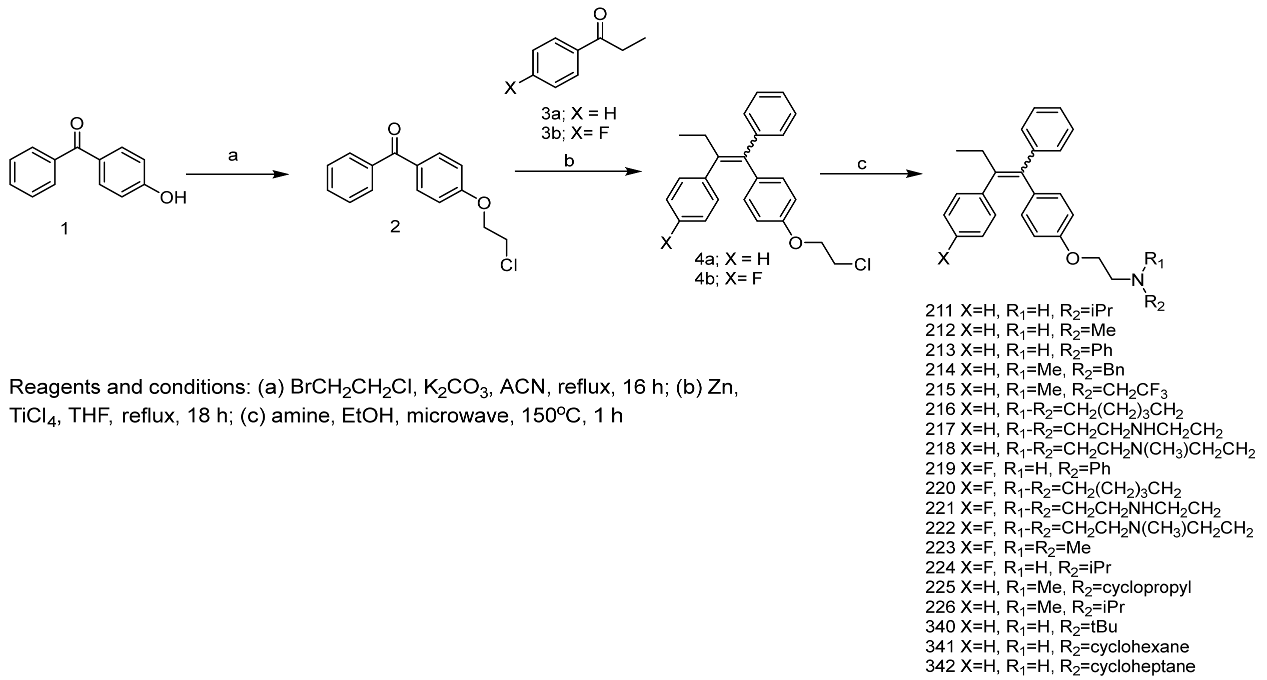

Figure S1. Synthesis of compounds 211–226 and 340–342.

Experimental Section

Synthesis of (4-(2-chloroethoxy)phenyl)(phenyl)methanone (2)[1]

To a suspension of 4-hydroxybenzophenone (**1**, 5.0 g, 25.2 mmol) and 1-bromo-2-chloroethane (4.2 mL, 50.4 mmol) in dry acetonitrile (63 mL) was added K_2CO_3 (10.4 g, 75.6 mmol) under an inert atmosphere. The reaction mixture was heated under reflux for 16 h and cooled to atmosphere temperature. The filtrate was concentrated under reduced pressure and purified by Biotage flash chromatography (gradient elution, 0–20% EtOAc in hexanes) to afford compound **2** as a white solid (6.57 g, 25.2 mmol, 63%). ^1H NMR (400 MHz, $CDCl_3$) δ 7.82 (d, *J* = 8.9 Hz, 2H), 7.76–7.71 (m, 2H), 7.59–7.53 (m, 1H), 7.46 (t, *J* = 7.5 Hz, 2H), 6.97 (d, *J* = 8.9 Hz, 2H), 4.30 (t, *J* = 5.8 Hz, 2H), 3.84 (t, *J* = 5.8 Hz, 2H). The spectroscopic data were consistent with those available in the literature.

General Procedure A: McMurry Coupling to Synthesize Triphenylethylene Compounds[2]

Titanium tetrachloride (11.5 mmol) was added drop wise to a stirred suspension of Zn dust (23 mmol) in dry THF (19 mL) under an argon atmosphere at −10 °C, and this mixture was heated under reflux for 1.5 h to produce the titanium reagent. A solution of **2** (3.84 mmol) and ketone/aldehyde (3.84 mmol) in dry THF (26 mL) was added to the titanium reagent at 0 °C, and the reaction was allowed to proceed at reflux for 2 h. After cooling to 25 °C, the reaction mixture was poured into a 10% aqueous K_2CO_3 solution (50 mL), this mixture was stirred vigorously for 20 min, and the dispersed insoluble material was removed by vacuum filtration. The organic fraction was separated, the aqueous layer was extracted with EtOAc (3 × 50 mL), and the combined organic fractions were dried (Na_2SO_4). The crude solution was concentrated under reduced pressure and purified by Biotage flash chromatography (gradient elution, 0–10% EtOAc in hexanes) to afford the triphenylethylene compound**.**

(1-(4-(2-chloroethoxy)phenyl)but-1-ene-1,2-diyl)dibenzene (4a)

According to general procedure A, titanium tetrachloride (5.0 mL, 45.9 mmol), Zn dust (6.0 g, 91.8 mmol) in THF (76.5 mL), compound **2** (4.0 g, 15.3 mmol), and propiophenone (**3a**, 2.0 mL, 15.3 mmol) in THF (102 mL) generated the title compound **4a** as a white solid, *E*/*Z* =1:3.1 (4.3 g, 11.9 mmol, 78%). (*Z*)-**4a**; ^{1}H NMR (400 MHz, $CDCl_3$) δ 7.33 (t, *J* = 7.3 Hz, 2H), 7.27 – 7.20 (m, 3H), 7.19 – 7.08 (m, 5H), 6.77 (d, *J* = 8.8 Hz, 2H), 6.54 (d, *J* = 8.8 Hz, 2H), 4.08 (t, *J* = 5.9 Hz, 2H), 3.71 (t, *J* = 5.9 Hz, 2H), 2.44 (q, *J* = 7.4 Hz, 2H), 0.91 (t, *J* = 7.5 Hz, 3H); (*E*)-**4a**; ^{1}H NMR (400 MHz, $CDCl_3$) δ 7.20–6.99 (m, 9H), 6.90–6.78 (m, 4H), 4.24 (t, *J* = 5.9 Hz, 2H), 3.81 (t, *J* = 5.9 Hz, 2H), 2.47 (q, *J* = 7.4 Hz, 2H), 0.93 (t, *J* = 7.5 Hz, 3H).

1. -(2-chloroethoxy)-4-(2-(4-fluorophenyl)-1-phenylbut-1-en-1-yl)benzene (4b)

According to general procedure A, titanium tetrachloride (0.63 mL, 5.76 mmol), Zn dust (753 mg, 11.5 mmol) in THF (9.6 mL), compound **2** (500 mg, 1.92 mmol), and 4′-fluoropropiophenone (**3b**, 0.27 mL, 1.92 mmol) in THF (12.8 mL) generated the title compound **4b** as a white solid, *E*/*Z* =1:3.6 (186 mg, 0.49 mmol, 25%). (*Z*)-**4b**; ^{1}H NMR (400 MHz, $CDCl_3$) δ 7.35 (t, *J* = 7.3 Hz, 2H), 7.31–7.21 (m, 3H), 7.09 (m, 2H), 6.93–6.83 (m, 2H), 6.80 (d, *J* = 8.7 Hz, 2H), 6.58 (d, *J* = 8.7 Hz, 2H), 4.09 (t, *J* = 5.9 Hz, 2H), 3.72 (t, *J* = 5.9 Hz, 2H), 2.46 (q, *J* = 7.5 Hz, 2H), 0.93 (t, *J* = 7.4 Hz, 3H). (*E*)-**4b**; ^{1}H NMR (400 MHz, $CDCl_3$) δ 7.21–6.99 (m, 9H), 6.93–6.83 (m, 4H), 4.24(t, *J* = 5.9 Hz, 2H), 3.81 (t, *J* = 5.9 Hz, 2H), 2.50 (q, *J* = 7.5 Hz, 2H), 0.95 (t, *J* = 7.4 Hz, 3H).

General Procedure B: Amination of Triphenylethylene Compounds

A mixture of **4** (0.083 mmol) and an appropriate amine (4.13 mmol) in 2-propanol (1.25 mL) was stirred at 150 °C for 1 h in a microwave reactor. Then, it was concentrated under reduced pressure and the crude was purified by Biotage flash chromatography (gradient elution, 0–20% EtOAc in hexanes) to obtain the triphenylethylene amines**.**

N-(2-(4-(1,2-diphenylbut-1-en-1-yl)phenoxy)ethyl)propan-2-amine (211)

According to general procedure B, compound **4a** (30 mg, 0.083 mmol), isopropylamine (0.34 mL, 4.13 mmol), and 2-propanol (1.3 mL) generated the title compound **211** as a white solid, *E*/*Z* =1:3.5 (13 mg, 0.034 mmol, 42%). The geometric isomers were separated using an HPLC equipped with a Diode Array Detector. A CHIRALPAK® AD-H Si gel column (Chiral Technologies Inc., Daicel group, 5 ◎m, 10 x 250 mm) was used with 1% IPA in hexane at a flow rate of 5 mL/min. The retention times of the isomers were 8.5 min (*E*) and 9.1 min (*Z*).

(E)-N-(2-(4-(1,2-diphenylbut-1-en-1-yl)phenoxy)ethyl)propan-2-amine (383)

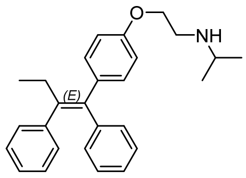

^{1}H NMR (400 MHz, $CDCl_3$) δ 7.13 (t, *J* = 7.3 Hz, 4H), 7.10 – 7.05 (m, 3H), 7.00 – 6.93 (m, 3H), 6.90 – 6.81 (m, 4H), 4.08 (t, *J* = 5.2 Hz, 2H), 3.00 (t, *J* = 5.2 Hz, 2H), 2.95 – 2.84 (m, 1H), 2.48 (q, *J* = 7.3 Hz, 2H), 1.10 (d, *J* = 6.2 Hz, 6H), 0.93 (t, *J* = 7.1 Hz, 3H); ^{13}C NMR (100 MHz, $CDCl_3$) δ 157.6, 143.3, 142.4, 141.9, 138.3, 136.2, 130.8, 130.5, 129.7, 127.7, 127.2, 126.0, 125.6, 114.1, 67.5, 48.5, 46.4, 29.7, 23.2, 13.5; HRMS (EI): m/z calculated for $C_{27}H_{31}NO$ $[M+1]^+$: 386.2478; found, 386.2478.

(Z)-N-(2-(4-(1,2-diphenylbut-1-en-1-yl)phenoxy)ethyl)propan-2-amine (384)

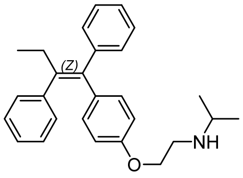

^{1}H NMR (400 MHz, $CDCl_3$) δ 7.33 (t, *J* = 7.3 Hz, 2H), 7.28 – 7.20 (m, 3H), 7.19 – 7.06 (m, 5H), 6.75 (d, *J* = 8.7 Hz, 2H), 6.53 (d, *J* = 8.7 Hz, 2H), 3.92 (t, *J* = 5.2 Hz, 2H), 2.90 (t, *J* = 5.2 Hz, 2H), 2.87 – 2.77 (m, 1H), 2.44 (q, *J* = 7.4 Hz, 2H), 1.05 (d, *J* = 6.2 Hz, 6H), 0.91 (t, *J* = 7.4 Hz, 3H); ^{13}C NMR (100 MHz, $CDCl_3$) δ 156.7, 143.8, 142.4, 141.3, 138.2, 135.6, 131.8, 129.7, 129.4, 128.1, 127.8, 126.5, 126.0, 113.4, 67.2, 48.5, 46.3, 29.0, 22.7, 13.6; HRMS (EI): m/z calcd for $C_{27}H_{31}NO$ $[M+1]^+$: 386.2478; found, 386.2478.

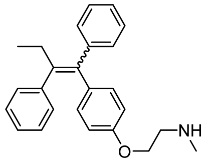

2. -(4-(1,2-diphenylbut-1-en-1-yl)phenoxy)-N-methylethan-1-amine (212)[3]

According to general procedure B, compound **4a** (29 mg, 0.08 mmol), methylamine (2.0 M in THF, 2 mL, 4 mmol), and 2-propanol (1.1 mL) generated the title compound **212** as a brown solid, *E*/*Z* =1:3 (24 mg, 0.067 mmol, 85%). (*Z*)-**212**; ^{1}H NMR (400 MHz, $CDCl_3$) δ 7.30 (t, *J* = 7.4 Hz, 2H), 7.25–7.16 (m, 3H), 7.16–7.04 (m, 5H), 6.76 (d, *J* = 8.5 Hz, 2H), 6.57 (d, *J* = 8.6 Hz, 2H), 4.10 (t, *J* = 4.6 Hz, 2H), 3.18 (t, *J* = 4.6 Hz, 2H), 2.57 (s, 3H), 2.41 (q, *J* = 7.4 Hz, 2H), 0.89 (t, *J* = 7.3 Hz, 3H). (*E*)-**212**; ^{1}H NMR (400 MHz, $CDCl_3$) δ 7.15–7.05 (m, 7H), 6.97–6.89 (m, 3H), 6.87–6.79 (m, 4H), 4.28 (t, *J* = 4.6 Hz, 2H), 3.29 (t, *J* = 4.6 Hz, 2H), 2.64 (s, 3H), 2.44 (q, *J* = 7.4 Hz, 2H), 0.91 (t, *J* = 7.3 Hz, 3H); HRMS (EI): m/z calculated for $C_{25}H_{27}NO$ $[M+1]^+$: 358.2165; found, 358.2164. The spectroscopic data were consistent with those available in the literature.

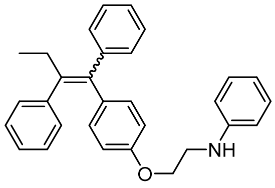

N-(2-(4-(1,2-diphenylbut-1-en-1-yl)phenoxy)ethyl)aniline (213)

According to general procedure B, compound **4a** (30 mg, 0.083 mmol), aniline (0.38 mL, 4.2 mmol), and 2-propanol (1.2 mL) generated the title compound **213** as an orange solid, *E*/*Z* =1:2.8 (27 mg, 0.075 mmol, 80%). (*Z*)-**213**; ^{1}H NMR (400 MHz, $CDCl_3$) δ 7.34 (t, *J* = 7.3 Hz, 3H), 7.30–7.21 (m, 5H), 7.20–7.07 (m, 5H), 6.82–6.76 (m, 3H), 6.58–6.52 (m, 3H), 4.08 (t, *J* = 5.9 Hz, 2H), 3.71 (t, *J* = 5.9 Hz, 2H), 2.46 (q, *J* = 7.5 Hz, 2H), 0.92 (t, *J* = 7.4 Hz, 3H). (*E*)-**213**; ^{1}H NMR (400 MHz, $CDCl_3$) δ 7.13–7.07 (m, 7H), 7.02–6.95 (m, 4H), 6.93–6.85 (m, 4H), 6.75–6.65 (m, 1H), 6.62 (m, 2H), 4.24 (t, *J* = 5.9 Hz, 2H), 3.80 (t, *J* = 5.9 Hz, 2H), 2.50 (q, *J* = 7.5 Hz, 2H), 0.94 (t, *J* = 7.4 Hz, 3H). HRMS (EI): m/z calculated for $C_{30}H_{29}NO$ $[M+1]^+$: 420.2322; found, 420.2319.

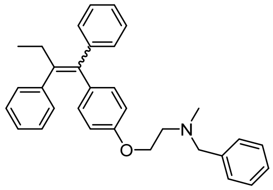

N-benzyl-2-(4-(1,2-diphenylbut-1-en-1-yl)phenoxy)-N-methylethan-1-amine (214)

According to general procedure B, compound **4a** (31 mg, 0.085 mmol), *N*-benzylmethylamine (0.38 mL, 4.2 mmol), and 2-propanol (1.2 mL) generated the title compound **214** as a colorless oil, *E*/*Z* =1:3 (27 mg, 0.06 mmol, 71%). (*Z*)- **214**; ^{1}H NMR (400 MHz, $CDCl_3$) δ 7.40 – 7.06 (m, 15H), 6.75 (d, *J* = 7.7 Hz, 2H), 6.52 (d, *J* = 7.7 Hz, 2H), 3.95 (t, *J* = 5.2 Hz, 2H), 3.56 (s, 2H), 2.74 (t, *J* = 5.1 Hz, 2H), 2.45 (q, *J* = 7.1 Hz, 2H), 2.28 (s, 3H), 0.92 (t, *J* = 7.3 Hz, 3H). (*E*)- **214**; ^{1}H NMR (400 MHz, $CDCl_3$) δ 7.40–7.06 (m, 12H), 6.98 (m, 3H), 6.86 (m, 4H), 4.11 (t, *J* = 5.2 Hz, 2H), 3.63 (s, 2H), 2.85 (t, *J* = 5.1 Hz, 2H), 2.50 (q, *J* = 7.1 Hz, 2H), 2.35 (s, 3H), 0.94 (t, *J* = 7.3 Hz, 3H); HRMS (EI): m/z calculated for $C_{32}H_{33}NO$ $[M+1]^+$: 448.2635; found, 448.2635.

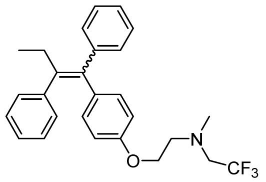

N-(2-(4-(1,2-diphenylbut-1-en-1-yl)phenoxy)ethyl)-2,2,2-trifluoro-N-methylethan-1-amine (215)[4]

According to general procedure B, compound **4a** (32 mg, 0.088 mmol), *N*-methyl-2,2,2-trifluoroethylamine (0.45 mL, 4.4 mmol), and 2-propanol (1.3 mL) generated the title compound **215** as a white solid, *E*/*Z* =1:3 (10 mg, 0.02 mmol, 26%). (*Z*)- **215**; ^{1}H NMR (400 MHz, $CDCl_3$) δ 7.33 (t, *J* = 7.4 Hz, 2H), 7.28–7.20 (m, 3H), 7.20–7.05 (m, 5H), 6.76 (d, *J* = 8.6 Hz, 2H), 6.52 (d, *J* = 8.6 Hz, 2H), 3.93 (t, *J* = 5.7 Hz, 2H), 3.12 (q, *J* = 9.5 Hz, 2H), 2.94 (t, *J* = 5.6 Hz, 2H), 2.49 (s, 3H), 2.43 (q, *J* = 7.4 Hz, 2H), 0.91 (t, *J* = 7.6 Hz, 3H). (*E*)- **215**; ^{1}H NMR (400 MHz, $CDCl_3$) δ 7.14–7.05 (m, 7H), 6.98 (m, 3H), 6.86 (m, 4H), 4.09 (t, *J* = 5.7 Hz, 2H), 3.20 (q, *J* = 9.5 Hz, 2H), 3.04 (t, *J* = 5.6 Hz, 2H), 2.57 (s, 3H), 2.47 (q, *J* = 7.4 Hz, 2H), 0.93 (t, *J* = 7.6 Hz, 3H); HRMS (EI): m/z calculated for $C_{27}H_{28}F_3NO$ $[M+1]^+$: 440.2196; found, 440.2187. The spectroscopic data were consistent with those available in the literature.

1. -(2-(4-(1,2-diphenylbut-1-en-1-yl)phenoxy)ethyl)piperidine (216)[5]

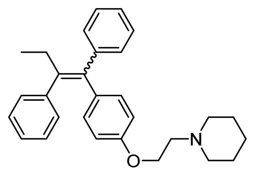

According to general procedure B, compound **4a** (29 mg, 0.08 mmol), piperidine (0.4 mL, 4 mmol), and 2-propanol (1.1 mL) generated the title compound **216** as a white solid, *E*/*Z* =1:2.5 (26 mg, 0.06 mmol, 79%). (*Z*)- **216**; ^{1}H NMR (400 MHz, $CDCl_3$) δ 7.33 (t, *J* = 7.3 Hz, 2H), 7.29–7.20 (m, 3H), 7.20–7.06 (m, 5H), 6.75 (d, *J* = 8.6 Hz, 2H), 6.53 (d, *J* = 8.6 Hz, 2H), 4.00 (t, *J* = 5.9 Hz, 2H), 2.73 (t, *J* = 5.7 Hz, 2H), 2.55–2.48 (m, 4H), 2.44 (q, *J* = 7.4 Hz, 2H), 1.64–1.56 (m, 4H), 1.51–1.37 (m, 2H), 0.91 (t, *J* = 7.6 Hz, 3H). (*E*)- **216**; ^{1}H NMR (400 MHz, $CDCl_3$) δ 7.13–7.06 (m, 7H), 7.02–6.94 (m, 3H), 6.90–6.84 (m, 4H), 4.15 (t, *J* = 5.9 Hz, 2H), 2.84 (t, *J* = 5.7 Hz, 2H), 2.63–2.55 (m, 4H), 2.47 (q, *J* = 7.4 Hz, 2H), 1.70–1.61 (m, 4H), 1.51–1.41 (m, 2H), 0.93 (t, *J* = 7.6 Hz, 3H). HRMS (EI): m/z calculated for $C_{29}H_{33}NO$ [M+1]$^+$: 412.2535; found, 412.2630. The spectroscopic data were consistent with those available in the literature.

1. -(2-(4-(1,2-diphenylbut-1-en-1-yl)phenoxy)ethyl)piperazine (217)

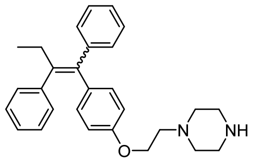

According to general procedure B, compound **4a** (32 mg, 0.088 mmol), piperazine (379 mg, 4.4 mmol), and 2-propanol (1.3 mL) generated the title compound **217** as an off-white solid, *E*/*Z* =1:2.6 (32 mg, 0.079 mmol, 89%). (*Z*)- **217**; ^{1}H NMR (400 MHz, $CDCl_3$) δ 7.32 (t, *J* = 7.3 Hz, 2H), 7.27–7.19 (m, 3H), 7.19–7.04 (m, 5H), 6.75 (d, *J* = 8.6 Hz, 2H), 6.50 (d, *J* = 8.7 Hz, 2H), 3.92 (t, *J* = 5.2 Hz, 2H), 3.19–3.11 (m, 4H), 2.84–2.76 (m, 4H), 2.74 (t, *J* = 5.1 Hz, 2H), 2.43 (q, *J* = 7.4 Hz, 2H), 0.90 (t, *J* = 7.6 Hz, 3H). (*E*)- **217**; ^{1}H NMR (400 MHz, $CDCl_3$) δ 7.14–7.04 (m, 7H), 7.01–6.94 (m, 3H) 6.87–6.81 (m, 4H), 4.07 (t, *J* = 5.2 Hz, 2H), 3.24–3.18 (m, 4H), 2.90–2.83 (m, 4H), 2.77 (t, *J* = 5.1 Hz, 2H), 2.47 (q, *J* = 7.4 Hz, 2H), 0.92 (t, *J* = 7.6 Hz, 3H). HRMS (EI): m/z calculated for $C_{28}H_{32}N_2O$ [M+1]$^+$: 413.2587; found, 413.2585.

1. -(2-(4-(1,2-diphenylbut-1-en-1-yl)phenoxy)ethyl)-4-methylpiperazine (218)[5]

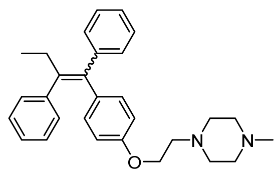

According to general procedure B, compound **4a** (31 mg, 0.085 mmol), 1-methylpiperazine (0.47 mL, 4.3 mmol), and 2-propanol (1.2 mL) generated the title compound **218** as an off-white solid, *E*/*Z* =1:3 (30 mg, 0.07 mmol, 83%). (*Z*)- **218**; ^{1}H NMR (400 MHz, $CDCl_3$) δ 7.32 (t, *J* = 7.3 Hz, 2H), 7.29–7.19 (m, 3H), 7.19–7.04 (m, 5H), 6.75 (d, *J* = 8.5 Hz, 2H), 6.52 (d, *J* = 8.5 Hz, 2H), 3.95 (t, *J* = 5.7 Hz, 2H), 2.72 (t, *J* = 5.8 Hz, 2H), 2.65–2.47 (m, 8H), 2.43 (q, *J* = 7.5 Hz, 2H), 2.30 (s, 3H), 0.90 (t, *J* = 7.6 Hz, 3H). (*E*)- **218**; ^{1}H NMR (400 MHz, $CDCl_3$) δ 7.15–7.05 (m, 7H), 7.01–6.93 (m, 3H), 6.88–6.83 (m, 4H), 4.10 (t, *J* = 5.7 Hz, 2H), 2.82 (t, *J* = 5.8 Hz, 2H), 2.67–2.49 (m, 8H), 2.45 (q, *J* = 7.5 Hz, 2H), 2.32 (s, 3H), 0.92 (t, *J* = 7.6 Hz, 3H); HRMS (EI): m/z calculated for $C_{29}H_{34}N_2O$ [M+1]$^+$: 427.2744; found, 427.2742. The spectroscopic data were consistent with those available in the literature.

N-benzyl-2-(4-(2-(4-fluorophenyl)-1-phenylbut-1-en-1-yl)phenoxy)-N-methylethan-1-amine (219)

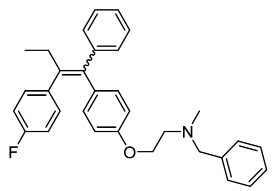

According to general procedure B, compound **4b** (24 mg, 0.063 mmol), *N*-benzylmethylamine (0.41 mL, 3.2 mmol) and 2-propanol (0.92 mL) generated the title compound **219** as a colorless oil, *E*/*Z* =1:3.6 (18 mg, 0.039 mmol, 61%). (*Z*)- **219**; ^{1}H NMR (400 MHz, $CDCl_3$) δ 7.38–7.19 (m, 10H), 7.10–7.03 (m, 2H), 6.90–6.81 (m, 2H), 6.73 (d, *J* = 8.7 Hz, 2H), 6.54 (d, *J* = 8.7 Hz, 2H), 3.95 (t, *J* = 6.0 Hz, 2H), 3.57 (s, 2H), 2.75 (t, *J* = 6.0 Hz, 2H), 2.43 (q, *J* = 7.4 Hz, 2H), 2.28 (s, 3H), 0.91 (t, *J* = 7.5 Hz, 3H). (*E*)- **219**; ^{1}H NMR (400 MHz, $CDCl_3$) δ 7.26–7.19 (4H), 7.14–6.97 (m, 10H), 6.89–6.82 (m, 4H), 4.10 (t, *J* = 6.0 Hz, 2H), 3.62 (s, 2H), 2.84 (t, *J* = 6.0 Hz, 2H), 2.48 (q, *J* = 7.4 Hz, 2H), 2.34 (s, 3H), 0.93 (t, *J* = 7.5 Hz, 3H). HRMS (EI): m/z calculated for $C_{32}H_{32}FNO$ [M+1]$^+$: 466.2541; found, 466.2541.

1. -(2-(4-(2-(4-fluorophenyl)-1-phenylbut-1-en-1-yl)phenoxy)ethyl)piperidine (220)

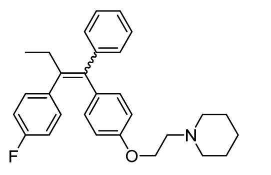

According to general procedure B, compound **4b** (24 mg, 0.063 mmol), piperidine (0.32 mL, 3.2 mmol) and 2-propanol (0.92 mL) generated the title compound **220** as a tan solid, *E*/*Z* =1:4 (21 mg, 0.049 mmol, 78%). (*Z*)- **220**; ^{1}H NMR (400 MHz, $CDCl_3$) δ 7.33 (t, *J* = 7.2 Hz, 2H), 7.29–7.19 (m, 3H), 7.10–7.04 (m, 2H), 6.86 (t, *J* = 8.5 Hz, 2H), 6.74 (d, *J* = 7.6 Hz, 2H), 6.56 (d, *J* = 8.0 Hz, 2H), 3.97 (t, *J* = 5.7 Hz, 2H), 2.69 (t, *J* = 5.7 Hz, 2H), 2.49–2.36 (m, 6H), 1.67–1.51 (m, 4H), 1.51–1.35 (m, 2H), 0.91 (t, *J* = 7.3 Hz, 3H). (*E*)- **220**; ^{1}H NMR (400 MHz, $CDCl_3$) δ 7.16–6.96 (m, 9H), 6.88–6.80 (m, 4H), 4.11 (t, *J* = 5.7 Hz, 2H), 2.78 (t, *J* = 5.7 Hz, 2H), 2.55–2.44 (m, 6H), 1.67–1.51 (m, 4H), 1.51–1.35 (m, 2H), 0.93 (t, *J* = 7.3 Hz, 3H); HRMS (EI): m/z calculated for $C_{29}H_{32}FNO$ [M+1]$^+$: 430.2541; found, 430.2541.

1. -(2-(4-(2-(4-fluorophenyl)-1-phenylbut-1-en-1-yl)phenoxy)ethyl)piperazine (221)

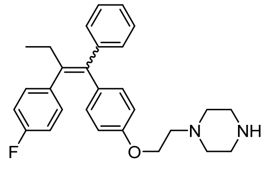

According to general procedure B, compound **4b** (22 mg, 0.058 mmol), piperazine (245 mg, 2.9 mmol), and 2-propanol (0.81 mL) generated the title compound **221** as a colorless oil, *E*/*Z* =1:4 (22 mg, 0.051 mmol, 88%). (*Z*)- **221**; ^{1}H NMR (400 MHz, $CDCl_3$) δ 7.34 (t, *J* = 7.1 Hz, 2H), 7.29–7.18 (m, 3H), 7.10–7.03 (m, 2H), 6.86 (t, *J* = 8.7 Hz, 2H), 6.75 (d, *J* = 7.7 Hz, 2H), 6.56 (d, *J* = 7.8 Hz, 2H), 3.98 (t, *J* = 5.6 Hz, 2H), 2.86 (m, 4H), 2.72 (t, *J* = 5.5 Hz, 2H), 2.50 (m, 4H), 2.43 (q, *J* = 7.2 Hz, 2H), 0.91 (t, *J* = 7.4 Hz, 3H). (*E*)- **221**; ^{1}H NMR (400 MHz, $CDCl_3$) δ 7.16–6.96 (m, 9H), 6.89–6.80 (m, 4H), 4.12 (t, *J* = 5.6 Hz, 2H), 2.91 (m, 4H), 2.81 (t, *J* = 5.5 Hz, 2H), 2.57 (m, 4H), 2.46 (q, *J* = 7.2 Hz, 2H), 0.93 (t, *J* = 7.4 Hz, 3H). HRMS (EI): m/z calculated for $C_{28}H_{31}FN_2O$ [M+1]$^+$: 431.2493; found, 431.2487.

1. -(2-(4-(2-(4-fluorophenyl)-1-phenylbut-1-en-1-yl)phenoxy)ethyl)-4-methylpiperazine (222)

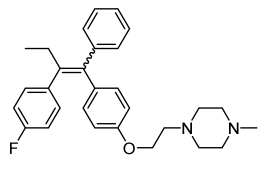

According to general procedure B, compound **4b** (28 mg, 0.073 mmol), methylpiperazine (0.41 mL, 3.7 mmol), and 2-propanol (1 mL) generated the title compound **222** as a brown solid, *E*/*Z* =1:3.4 (22 mg, 0.055 mmol, 75%). (*Z*)- **222**; ^{1}H NMR (400 MHz, $CDCl_3$) δ 7.34 (t, *J* = 6.9 Hz, 2H), 7.29–7.18 (m, 3H), 7.10–7.04 (m, 2H), 6.86 (t, *J* = 8.3 Hz, 2H), 6.75 (d, *J* = 7.8 Hz, 2H), 6.56 (d, *J* = 7.8 Hz, 2H), 3.97 (t, *J* = 5.2 Hz, 2H), 2.73 (t, *J* = 5.2 Hz, 2H), 2.63–2.37 (m, 10H), 2.28 (s, 3H), 0.91 (t, *J* = 7.3 Hz, 3H). (*E*)- **222**; ^{1}H NMR (400 MHz, $CDCl_3$) δ 7.16–6.97 (m, 9H), 6.88–6.79 (m, 4H), 4.12 (t, *J* = 5.2 Hz, 2H), 2.83 (t, *J* = 5.2 Hz, 2H), 2.63–2.37 (m, 10H), 2.30 (s, 3H), 0.93 (t, *J* = 7.3 Hz, 3H); HRMS (EI): m/z calculated for $C_{29}H_{33}FN_2O$ [M+1]$^+$: 445.2650; found, 445.2650.

2. -(4-(2-(4-fluorophenyl)-1-phenylbut-1-en-1-yl)phenoxy)-N,N-dimethylethan-1-amine (223)

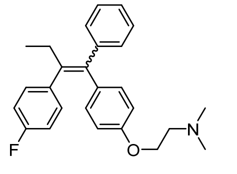

According to general procedure B, compound **4b** (23 mg, 0.06 mmol) and dimethylamine (2.0 M in THF, 1.5 mL, 3 mmol) generated the title compound **223** as a tan solid, *E*/*Z* =1:3.4 (6.4 mg, 0.016 mmol, 27%). (*Z*)- **223**; ^{1}H NMR (400 MHz, $CDCl_3$) δ 7.34 (t, *J* = 7.3 Hz, 2H), 7.29–7.19 (m, 3H), 7.11–7.04 (m, 2H), 6.86 (t, *J* = 8.7 Hz, 2H), 6.75 (d, *J* = 8.7 Hz, 2H), 6.57 (d, *J* = 8.7 Hz, 2H), 3.97 (t, *J* = 5.7 Hz, 2H), 2.71 (t, *J* = 5.6 Hz, 2H), 2.43 (q, *J* = 7.1 Hz, 2H), 2.34 (s, 6H), 0.91 (t, *J* = 7.5 Hz, 4H). (*E*)- **223**; ^{1}H NMR (400 MHz, $CDCl_3$) δ 7.16–6.98 (m, 9H), 6.91–6.81 (m, 4H), 4.11 (t, *J* = 5.7 Hz, 2H), 2.80 (t, *J* = 5.6 Hz, 2H), 2.48 (q, *J* = 7.1 Hz, 2H), 2.39 (s, 6H), 0.94 (t, *J* = 7.5 Hz, 4H); HRMS (EI): m/z calculated for $C_{26}H_{28}FNO$ [M+1]$^+$: 390.2228; found, 390.2216.

N-(2-(4-(2-(4-fluorophenyl)-1-phenylbut-1-en-1-yl)phenoxy)ethyl)propan-2-amine (224)

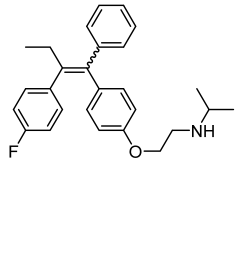

According to general procedure B, compound **4b** (23 mg, 0.06 mmol), isopropylamine (0.25 mL, 3 mmol) and 2-propanol (0.85 mL) generated the title compound **224** as a white solid, *E*/*Z* = 1:3.7 (11.5 mg, 0.028 mmol, 48%). (*Z*)- **224**; ^{1}H NMR (400 MHz, $CDCl_3$) δ 7.31 (t, *J* = 7.4 Hz, 2H), 7.27–7.17 (m, 3H), 7.08–7.01 (m, 2H), 6.84 (t, *J* = 8.6 Hz, 2H), 6.73 (d, *J* = 8.7 Hz, 2H), 6.55 (d, *J* = 8.7 Hz, 2H), 3.99 (t, *J* = 5.2 Hz, 2H), 2.97 (t, *J* = 5.0 Hz, 2H), 2.41 (q, *J* = 7.5 Hz, 2H), 1.13 (d, *J* = 6.3 Hz, 6H), 0.90 (t, *J* = 7.3 Hz, 3H). (*E*)- **224**; ^{1}H NMR (400 MHz, $CDCl_3$) δ 7.14–6.94 (m, 9H), 6.90–6.78 (m, 4H), 4.13 (t, *J* = 5.2 Hz, 2H), 3.05 (t, *J* = 5.0 Hz,

2H), 2.46 (q, *J* = 7.5 Hz, 2H), 1.18 (d, *J* = 6.3 Hz, 6H), 0.92 (t, *J* = 7.3 Hz, 3H). HRMS (EI): m/z calculated for $C_{27}H_{30}FNO$ [M+1]$^+$: 404.2384; found, 404.2371.

N-(2-(4-(1,2-diphenylbut-1-en-1-yl)phenoxy)ethyl)-N-methylcyclopropanamine (225)

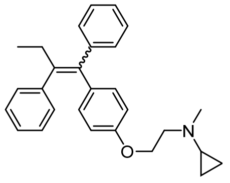

According to general procedure B, compound **4a** (27 mg, 0.074 mmol), *N*-methylcyclopropanamine (0.31 mL, 3.7 mmol), and 2-propanol (1.1 mL) generated the title compound **225** as a tan solid, *E*/*Z* =1:2.9 (13 mg, 0.033 mmol, 45%). (*Z*)- **225**; ^{1}H NMR (400 MHz, $CDCl_3$) δ 7.34 (t, *J* = 7.3 Hz, 2H), 7.30–7.21 (m, 3H), 7.20–7.06 (m, 5H), 6.76 (d, *J* = 8.7 Hz, 2H), 6.54 (d, *J* = 8.7 Hz, 2H), 3.96 (t, *J* = 6.1 Hz, 2H), 2.87 (t, *J* = 6.1 Hz, 2H), 2.45 (q, *J* = 7.6 Hz, 2H), 2.39 (s, 3H), 1.71–1.63 (m, 1H), 0.92 (t, *J* = 7.5 Hz, 3H), 0.47–0.36 (m, 4H). (*E*)- **225**; ^{1}H NMR (400 MHz, $CDCl_3$) δ 7.14–7.07 (m, 7H), 7.02–6.94 (m, 3H), 6.91–6.85 (m, 4H), 4.12 (t, *J* = 6.1 Hz, 2H), 2.98 (t, *J* = 6.1 Hz, 2H), 2.51 (q, *J* = 7.6 Hz, 2H), 2.46 (s, 3H), 1.74 (m, 1H), 0.94 (t, *J* = 7.5 Hz, 3H), 0.53–0.42 (m, 4H); HRMS (EI): m/z calculated for $C_{28}H_{31}NO$ [M+1]$^+$: 398.2478; found, 398.2465.

N-(2-(4-(1,2-diphenylbut-1-en-1-yl)phenoxy)ethyl)-N-methylpropan-2-amine (226)

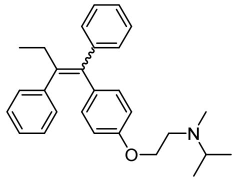

According to general procedure B, compound **4a** (29 mg, 0.08 mmol), *N*-methylisopropylamine (0.42 mL, 4 mmol), and 2-propanol (1.1 mL) generated the title compound **226** as a colorless oil, *E*/*Z* = 1:2.9 (18 mg, 0.046 mmol, 57%). (*Z*)- **226**; ^{1}H NMR (400 MHz, $CDCl_3$) δ 7.33 (t, *J* = 7.3 Hz, 2H), 7.28–7.20 (m, 3H), 7.19–7.05 (m, 5H), 6.75 (d, *J* = 8.7 Hz, 2H), 6.53 (d, *J* = 8.7 Hz, 2H), 3.97 (t, *J* = 6.1 Hz, 2H), 2.99–2.92 (m, 1H), 2.78 (t, *J* = 6.1 Hz, 2H), 2.44 (q, *J* = 7.4 Hz, 2H), 2.33 (s, 3H), 1.04 (d, *J* = 6.6 Hz, 6H), 0.91 (t, *J* = 7.5 Hz, 3H). (*E*)- **226**; ^{1}H NMR (400 MHz, $CDCl_3$) δ 7.13–7.06 (m, 7H), 7.01–6.93 (m, 3H), 6.90–6.83 (m, 4H), 4.13 (t, *J* = 6.1 Hz, 2H), 3.01 (m, 1H), 2.88 (t, *J* = 6.1 Hz, 2H), 2.49 (q, *J* = 7.4 Hz, 2H), 2.38 (s, 3H), 1.09 (d, *J* = 6.6 Hz, 6H), 0.93 (t, *J* = 7.5 Hz, 3H). HRMS (EI): m/z calculated for $C_{28}H_{33}NO$ [M+1]$^+$: 400.2635; found, 400.2631.

N-(2-(4-(1,2-diphenylbut-1-en-1-yl)phenoxy)ethyl)-2-methylpropan-2-amine (340)

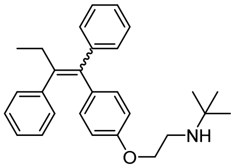

According to general procedure B, compound **4a** (30 mg, 0.083 mmol), *tert*-butylamine (0.43 mL, 4.2 mmol), and 2-propanol (0.83 mL) generated the title compound **340** as a white solid, *E*/*Z* =1:2.5 (15 mg, 0.037 mmol, 45%). (*Z*)- **340**; ^{1}H NMR (400 MHz, CD_3OD) δ 7.33 (t, *J* = 7.3 Hz, 2H), 7.21–7.17 (m, 2H), 7.14–7.05 (m, 6H), 6.80 (d, *J* = 8.8 Hz, 2H), 6.64 (d, *J* = 8.8 Hz, 2H), 4.09 (t, *J* = 5.0 Hz, 2H), 3.26 (t, *J* = 5.0 Hz, 2H), 2.43 (q, *J* = 7.3 Hz, 2H), 1.33 (s, 9H), 0.88 (t, *J* = 7.5 Hz, 3H). (*E*)- **340**; ^{1}H NMR (400 MHz, CD_3OD) δ 7.13–7.05 (m, 7H), 7.02–6.92 (m, 3H), 6.86–6.81 (m, 4H), 4.26 (t, *J* = 5.0 Hz, 2H), 3.36 (t, *J* = 5.0 Hz, 2H), 2.46 (q, *J* = 7.3 Hz, 2H), 1.38 (s, 9H), 0.90 (t, *J* = 7.5 Hz, 3H); HRMS (EI): m/z calculated for $C_{28}H_{33}NO$ [M+1]$^+$: 400.2535; found, 400.2629.

N-(2-(4-(1,2-diphenylbut-1-en-1-yl)phenoxy)ethyl)cyclohexanamine (341)

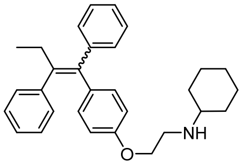

According to general procedure B, compound **4a** (30 mg, 0.083 mmol), cyclohexylamine (0.48 mL, 4.15 mmol), and 2-propanol (0.83 mL) generated the title compound **341** as a brownish oil, *E*/*Z* =1:3.3 (20 mg, 0.046 mmol, 56%). (*Z*)- **341**; ^{1}H NMR (400 MHz, CD_3OD) δ 7.31 (t, *J* = 7.3 Hz, 2H), 7.22–7.16 (m, 2H), 7.15 – 7.03 (m, 6H), 6.75 (d, *J* = 8.7 Hz, 2H), 6.56 (d, *J* = 8.8 Hz, 2H), 3.94 (t, *J* = 5.3 Hz, 2H), 2.95 (t, *J* = 5.3 Hz, 2H), 2.57–2.47 (m, 1H), 2.42 (q, *J* = 7.4 Hz, 2H), 2.02–1.57 (m, 4H), 1.34–1.04 (m, 6H), 0.88 (t, *J* = 7.6 Hz, 3H). (*E*)- **341**; 7.14–7.03 (m, 7H), 6.95–6.90 (m, 3H), 6.85–6.81 (m, 4H), 4.10 (t, *J* = 5.3 Hz, 2H), 3.05 (t, *J* = 5.3 Hz, 2H), 2.63–2.53 (m, 1H), 2.46 (q, *J* = 7.4 Hz, 2H), 2.02–1.57 (m, 4H), 1.34–1.04 (m, 6H), 0.90 (t, *J* = 7.6 Hz, 3H). HRMS (EI): m/z calculated for $C_{30}H_{35}NO$ [M+1]$^+$: 426.2791; found, 426.2786.

N-(2-(4-(1,2-diphenylbut-1-en-1-yl)phenoxy)ethyl)cycloheptanamine (342)

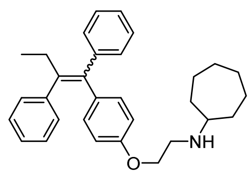

According to general procedure B, compound **4a** (30 mg, 0.083 mmol), cycloheptanamine (0.53 mL, 4.15 mmol), and 2-propanol (0.83 mL) generated the title compound **342** as a tan solid, *E*/*Z* =1:3 (34 mg, 0.077 mmol, 92%). (*Z*)- **342**; ^{1}H NMR (400 MHz, CD_3OD) δ 7.32 (t, *J* = 7.3 Hz, 2H), 7.22–7.16 (m, 2H), 7.14–7.02 (m, 6H), 6.80 (d, *J* = 8.8 Hz, 2H), 6.63 (d, *J* = 8.8 Hz, 2H), 4.11 (t, *J* = 5.0 Hz, 2H), 3.33 (t, *J* = 5.0 Hz, 2H), 3.27–3.18 (m, 1H), 2.43 (q, *J* = 7.4 Hz, 2H), 2.11–2.02 (m, 2H), 1.84–1.42 (m, 10H), 0.88 (t, *J* = 7.5 Hz, 3H). (*E*)- **342**; 7.13–7.03 (m, 7H), 7.02–6.92 (m, 3H), 6.86–6.81 (m, 4H), 4.28 (t, *J* = 5.0 Hz, 2H), 3.43 (t, *J* = 5.0 Hz, 2H), 3.27–3.18 (m, 1H), 2.46 (q, *J* = 7.4 Hz, 2H), 2.11–2.02 (m, 2H), 1.84–1.42 (m, 10H), 0.90 (t, *J* = 7.5 Hz, 3H). HRMS (EI): m/z calculated for $C_{31}H_{37}NO$ [M+1]$^+$: 440.2948; found, 440.2943.

Synthesis of Compounds 263–271 and 343

Experimental Section

2. -(4-(1,2-diphenylbutyl)phenoxy)-N,N-dimethylethan-1-amine (263)[6]

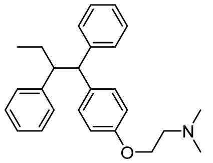

According to general procedure B, compound **4a** (32mg, 0.088 mmol), dimethylamine (2.0 M in THF, 2.2 mL, 4.4 mmol), and 2-propanol (1.3 mL) generated tamoxifen as a white solid (24 mg, 0.065 mmol, 72%). To a solution of tamoxifen (15 mg, 0.04 mmol) in methanol (0.4 mL) was added Pd/C (10% activated on charcoal, 4.3 mg, 0.004 mmol). The suspension was degassed and re-purged with hydrogen gas (this process was repeated three times). The reaction was stirred under hydrogen atmosphere at ambient temperature for 16 h. The reaction was filtered through Celite, washed repeatedly with DCM/MeOH, and concentrated under reduced pressure to generate the title compound **263** as colorless oil (13 mg, 0.035 mmol, 87%). Major isomer: ^{1}H NMR (400 MHz, $CDCl_3$) δ 7.30–7.21 (m, 2H), 7.19 – 6.96 (m, 11H), 6.85 (d, *J* = 8.4 Hz, 2H), 4.08–3.99 (m, 3H), 3.25 (t, *J* = 10.7 Hz, 1H), 2.70 (t, *J* = 5.7 Hz, 2H), 2.31 (s, 6H), 1.69 (m, 1H), 1.43 (m, 1H), 0.64 (t, *J* = 7.2 Hz, 3H). HRMS (EI): m/z calculated for $C_{26}H_{31}NO$ [M+1]$^+$: 374.2478; found, 374.2473. The spectroscopic data were consistent with those reported in the literature.

N,N-dimethyl-2-(4-(2-phenylbut-1-en-1-yl)phenoxy)ethan-1-amine (264)[7]

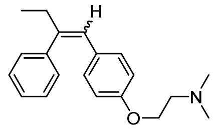

According to the synthesis of compound **2**, 4-hydroxybenzaldehyde (1.0 g, 8.2 mmol), 1-bromo-2-chloroethane (1.37 mL, 16.4 mmol), and K_2CO_3 (3.4 g, 25 mmol) in acetonitrile (20 mL) generated chloroethane-benzaldehyde intermediate as a yellowish oil (1.1 g, 6.0 mmol, 73%). According to general procedure A, titanium tetrachloride (0.44 mL, 4.05 mmol), Zn dust (530 mg, 8.1 mmol) in THF (6.8 mL), chloroethane-benzaldehyde intermediate (250 mg, 1.35 mmol), and propiophenone (**3a**, 0.18 mL, 1.35 mmol) in THF (9 mL) stirred overnight generated the diphenylethylene intermediate (41 mg, 0.14 mmol, 10%). According to general procedure B, diphenylethylene intermediate (41 mg, 0.14 mmol) and dimethylamine (2.0 M in THF, 2.0 mL, 4 mmol) generated the title compound **264** as a tan solid, *E*/*Z* =1.7:1 (24 mg, 0.08 mmol, 58%). (*E*)- **264**; ^{1}H NMR (400 MHz, CD_3OD) δ 7.42 (d, *J* = 7.2 Hz, 2H), 7.31 (t, *J* = 7.5 Hz, 2H), 7.25 (m, 3H), 6.95 (d, *J* = 8.7 Hz, 2H), 6.59 (s, 1H), 4.16 (t, *J* = 5.3 Hz, 2H), 2.96 (t, *J* = 5.3 Hz, 2H), 2.71 (q, *J* = 7.5 Hz, 2H), 2.49 (s, 6H), 1.00 (t, *J* = 7.5 Hz, 3H); (*Z*)- **264**; ^{1}H NMR (400 MHz, CD_3OD) δ 7.30–7.19 (m, 3H), 7.09 (d, *J* = 6.8 Hz, 2H), 6.81 (d, *J* = 8.6 Hz, 2H), 6.63 (d, *J* = 8.8 Hz, 2H), 6.36 (s, 1H), 4.03 (t, *J* = 5.3 Hz, 1H), 2.90 (t, *J* = 5.3 Hz, 1H), 2.71 (q, *J* = 7.5 Hz, 2H), 2.45 (s, 6H), 1.00 (t, *J* = 7.5 Hz, 3H); HRMS (EI): m/z calculated for $C_{20}H_{25}NO$ [M+1]$^+$: 296.2009; found, 296.2002. The spectroscopic data were consistent with those reported in the literature.

N,N-dimethyl-2-(4-(1-phenylbut-1-en-1-yl)phenoxy)ethan-1-amine (265)

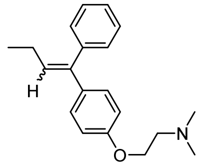

According to general procedure A, titanium tetrachloride (0.32 mL, 2.88 mmol), Zn dust (377 mg, 5.76 mmol) in THF (4.8 mL), compound **2** (250 mg, 0.96 mmol), and propanal (0.41mL, 5.76 mmol) in THF (6.4 mL) stirred overnight generated the diphenylethylene intermediate (197 mg, 0.69 mmol, 72%). According to general procedure B, diphenylethylene intermediate (41 mg, 0.14 mmol) and dimethylamine (2.0 M in THF, 1.43 mL, 2.86 mmol) generated the title compound **265** as a tan oil, *E*/*Z* =1:1.7 (32 mg, 0.11 mmol, 76%). (*Z*)- **265**; ^{1}H NMR (400 MHz, CD_3OD) δ 7.33 (t, *J* = 8.7 Hz, 2H), 7.14–7.06 (m, 5H), 6.84 (d, *J* = 8.7 Hz, 2H), 5.97 (m, 1H), 4.16 (t, *J* = 5.2 Hz, 2H), 3.12 (t, *J* = 5.2 Hz, 2H), 2.60 (s, 6H), 2.03 (m, 2H), 0.98(t, *J* = 7.3 Hz, 3H). (*E*)- **265**; ^{1}H NMR (400 MHz, CD_3OD) δ 7.29–7.12 (m, 5H), 7.05 (d, *J* = 8.6 Hz, 2H), 6.98 (d, *J* = 8.6 Hz, 2H), 6.00 (m, 1H), 4.22 (t, *J* = 5.2 Hz, 2H), 3.15 (t, *J* = 5.2 Hz, 2H), 2.63 (s, 6H), 2.08 (m, 2H), 1.00 (t, *J* = 7.3 Hz, 3H); HRMS (EI): m/z calculated for $C_{20}H_{25}NO$ [M+1]$^{+}$: 296.2009; found, 296.2005.

2. -(4-(1,2-diphenylvinyl)phenoxy)-N,N-dimethylethan-1-amine (266)[8]

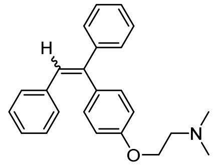

According to general procedure A, titanium tetrachloride (0.32 mL, 2.88 mmol), Zn dust (377 mg, 5.76 mmol) in THF (4.8 mL), compound **2** (250 mg, 0.96 mmol), and benzaldehyde (0.59 mL, 5.76 mmol) in THF (6.4 mL) stirred overnight generated the triphenylethylene intermediate (150 mg, 0.45 mmol, 65%). According to general procedure B, triphenylethylene intermediate (37 mg, 0.11 mmol) and dimethylamine (2.0 M in THF, 1.1 mL, 2.2 mmol) generated the title compound **266** as a tan oil, *E*/*Z* =1:1 (27 mg, 0.079 mmol, 70%). ^{1}H NMR (400 MHz, CD_3OD) δ 7.32–7.18 (m, 5H), 7.13–6.99 (m, 5H), 6.97–6.86 (m, 4H), 4.15 (m, 2H), 3.01 (m, 2H), 2.52 (s, 6H); HRMS (EI): m/z calculated for $C_{24}H_{25}NO$ [M+1]$^{+}$: 344.2009; found, 344.2004. The spectroscopic data were consistent with those reported in the literature.

6. -(4-(1,2-diphenylbut-1-en-1-yl)phenoxy)-N,N-dimethylhexan-1-amine (267)

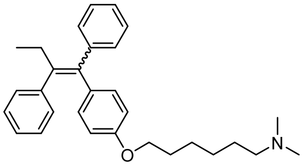

According to the synthesis of compound **2**, 4-hydroxybenzophenone (**1**, 2.0 g, 10.1 mmol), 1-bromo-6-chlorohexane (3 mL, 20 mmol), and K_2CO_3 (4.2 g, 30 mmol) in acetonitrile (25.3 mL) generated 6-chlorohexane-benzophenone intermediate (568 mg, 1.8 mmol, 18%). According to general procedure A, titanium tetrachloride (0.59 mL, 5.37 mmol), Zn dust (702 mg, 10.7 mmol) in THF (9.0 mL), 6-chlorohexane-benzophenone intermediate (568 mg, 1.8 mmol), and propiophenone (**3a**, 0.24 mL, 1.8 mmol) in THF (12 mL) stirred overnight generated the triphenylethylene intermediate (268 mg, 0.64 mmol, 36%). According to general procedure B, triphenylethylene intermediate (40 mg, 0.095 mmol) and dimethylamine (2.0 M in THF, 0.95 mL, 1.9 mmol) generated the title compound **267** as a yellowish oil, *E*/*Z* =1:3.5 (26 mg, 0.061 mmol, 64%). (*Z*)- **267**; ^{1}H NMR (400 MHz, $CDCl_3$) δ 7.32 (t, *J* = 7.3 Hz, 2H), 7.26–7.19 (m, 3H), 7.16–7.07 (m, 5H), 6.74 (d, *J* = 8.6 Hz, 2H), 6.49 (d, *J* = 8.6 Hz, 2H), 3.78 (t, *J* = 6.1 Hz, 2H), 3.00–2.94 (m, 2H), 2.73 (s, 6H), 2.42 (q, *J* = 7.4 Hz, 2H), 1.93–1.62 (m, 4H), 1.51–1.33 (m, 4H), 0.89 (t, *J* = 7.4 Hz, 3H). (*E*)- **267**; ^{1}H NMR (400 MHz, $CDCl_3$) δ 7.18–7.04 (m, 7H), 7.01–6.92 (m, 3H), 6.88–6.81 (m, 4H), 3.95 (t, *J* = 6.1 Hz, 2H), 3.05–2.98 (m, 2H), 2.75 (s, 6H), 2.47 (q, *J* = 7.4 Hz, 2H), 1.93–1.62 (m, 4H), 1.51–1.33 (m, 4H), 0.92 (t, *J* = 7.4 Hz, 3H); HRMS (EI): m/z calculated for $C_{30}H_{37}NO$ [M+1]$^{+}$: 428.2948; found, 428.2944.

2. ,2′-(((2-phenylbut-1-ene-1,1-diyl)bis(4,1-phenylene))bis(oxy))bis(N,N-dimethylethan-1-amine) (268)[9]

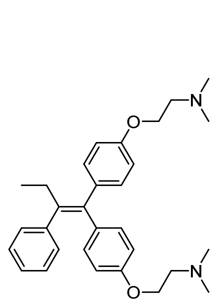

According to the synthesis of compound **2**, 4,4′-dihydroxybenzophenone (1.0 g, 4.67 mmol), 1-bromo-2-chloroethane (1.55 mL, 18.7 mmol), and K_2CO_3 (4.9 g, 28.1 mmol) in acetonitrile (23.4 mL) generated dichloroethane-benzophenone intermediate as an off white solid (870 mg, 12.6 mmol, 55%). According to general procedure A, titanium tetrachloride (0.24 mL, 2.22 mmol), Zn dust (292 mg, 4.46 mmol) in THF (3.7 mL), dichloroethane-benzophenone intermediate (250 mg, 0.74 mmol), and propiophenone (**3a**, 0.01

mL, 0.74 mmol) in THF (4.9 mL) stirred overnight generated the triphenylethylene intermediate (88 mg, 0.20 mmol, 27%). According to general procedure B, triphenylethylene intermediate (25 mg, 0.06 mmol) and dimethylamine (2.0 M in THF, 3 mL, 6.0 mmol) generated the title compound **268** as an orange solid (63 mg, 0.14 mmol, 98%). ^{1}H NMR (400 MHz, CD_3OD) δ 7.20–6.96 (m, 9H), 6.78 (d, *J* = 8.6 Hz, 2H), 6.67 (d, *J* = 8.6 Hz, 2H), 4.39 (t, *J* = 4.7 Hz, 2H), 4.21 (t, *J* = 4.7 Hz, 2H), 3.66 (t, *J* = 5.0 Hz, 2H), 3.56 (t, *J* = 5.0 Hz, 2H), 3.01 (s, 6H), 2.94 (s, 6H), 2.43 (q, *J* = 7.3 Hz, 2H), 0.88 (t, *J* = 7.4 Hz, 3H); HRMS (EI): m/z calculated for $C_{30}H_{38}N_2O_2$ $[M+2]^{2+}$: 230.1539; found, 230.1539. The spectroscopic data were consistent with those reported in the literature.

N,N-dimethyl-2-(4-(2-(4-(2-(dimethylamino)ethoxy)phenyl)-1-phenylbut-1-en-1-yl)phenoxy)ethan-1-amine (269)

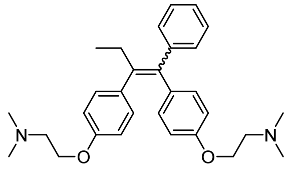

According to the synthesis of compound **2**, 4-hydroxypropiophenone (500 mg, 3.33 mmol), 1-bromo-2-chloroethane (0.55 mL, 6.66 mmol), and K_2CO_3 (1.4 g, 10 mmol) in acetonitrile (8.3 mL) generated chloroethane-propiophenone intermediate (419 mg, 2.0 mmol, 59%). According to general procedure A, titanium tetrachloride (0.12 mL, 1.14 mmol), Zn dust (149 mg, 2.28 mmol) in THF (1.9 mL), compound **2** (100 mg, 0.38 mmol) and chloroethane-propiophenone intermediate (81mg, 0.38 mmol) in THF (2.5 mL) stirred overnight generated the triphenylethylene intermediate 127 mg, 0.29 mmol, 76%). According to general procedure B, triphenylethylene intermediate (41 mg, 0.093 mmol) and dimethylamine (2.0 M in THF, 0.93 mL, 1.86 mmol) generated the title compound **269** as a yellowish oil, *E*/*Z* =1:3.7 (32 mg, 0.07 mmol, 75%). (*Z*)- **269**; ^{1}H NMR (400 MHz, CD_3OD) δ 7.30 (t, *J* = 7.3 Hz, 2H), 7.25–7.14 (m, 3H), 7.01 (d, *J* = 8.7 Hz, 2H), 6.74 (dd, *J* = 8.7, 7.4 Hz, 4H), 6.56 (d, *J* = 8.8 Hz, 2H), 4.02 (t, *J* = 5.5 Hz, 2H), 3.94 (t, *J* = 5.5 Hz, 2H), 2.75 (t, *J* = 5.4 Hz, 2H), 2.70 (t, *J* = 5.5 Hz, 2H), 2.37–2.32 (m, 8H), 2.30 (s, 6H), 0.88 (t, *J* = 7.5 Hz, 3H). (*E*)- **269**; ^{1}H NMR (400 MHz, CD_3OD) δ 7.12–7.07 (m, 4H), 6.99–6.87 (m, 3H), 6.86–6.80 (m, 4H), 6.69–6.60 (m, 2H), 4.09 (t, *J* = 5.5 Hz, 2H), 4.01 (t, *J* = 5.5 Hz, 2H), 2.79 (t, *J* = 5.4 Hz, 2H), 2.74 (t, *J* = 5.5 Hz, 2H), 2.44–2.37 (m, 2H), 2.36 (s, 6H), 2.31 (s, 6H), 0.91 (t, *J* = 7.5 Hz, 3H). HRMS (EI): m/z calculated for $C_{30}H_{38}N_2O_2$ $[M+2]^{2+}$: 230.1539; found, 230.1536.

2. -(4-(2-(4-chlorophenyl)-1-phenylbut-1-en-1-yl)phenoxy)-N,N-dimethylethan-1-amine (270)

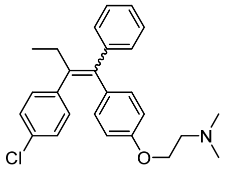

According to general procedure A, titanium tetrachloride (0.12 mL, 1.14 mmol), Zn dust (149 mg, 2.28 mmol) in THF (1.9 mL), compound **2** (100 mg, 0.38 mmol), and 4′-chloropropiophenone (65 mg, 0.38 mmol) in THF (2.5 mL) stirred overnight generated the 4′-chloro-triphenylethylene intermediate (106 mg, 0.27 mmol, 70%). According to general procedure B, 4′-chloro-triphenylethylene intermediate (40 mg, 0.1 mmol) and dimethylamine (2.0 M in THF, 1.0 mL, 2.0 mmol) generated the title compound **270** as a tan solid, *E*/*Z* =1:3.3 (25 mg, 0.065 mmol, 62%). (*Z*)- **270**; ^{1}H NMR (400 MHz, $CDCl_3$) δ 7.32 (t, *J* = 7.3 Hz, 2H), 7.28–6.97 (m, 5H), 6.74 (d, *J* = 8.7 Hz, 2H), 6.57 (d, *J* = 8.8 Hz, 2H), 4.00 (t, *J* = 5.6 Hz, 2H), 2.77 (t, *J* = 5.6 Hz, 2H), 2.45–2.40 (m, 2H), 2.38 (s, 6H), 0.89 (t, *J* = 7.4 Hz, 3H). (*E*)- **270**; ^{1}H NMR (400 MHz, $CDCl_3$) δ 7.27–6.98 (m, 9H), 6.91–6.82 (m, 4H), 4.13 (t, *J* = 5.6 Hz, 2H), 2.85 (t, *J* = 5.6 Hz, 2H), 2.47–2.43 (m, 2H), 2.41 (s, 6H), 0.92 (t, *J* = 7.4 Hz, 3H); HRMS (EI): m/z calculated for $C_{26}H_{28}ClNO$ $[M+1]^+$: 406.1932; found, 406.1928.

2. -(4-(2-(4-bromophenyl)-1-phenylbut-1-en-1-yl)phenoxy)-N,N-dimethylethan-1-amine (271)[10]

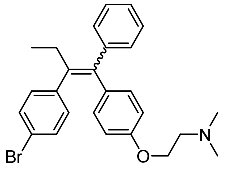

According to general procedure A, titanium tetrachloride (0.12 mL, 1.14 mmol), Zn dust (149 mg, 2.28 mmol) in THF (1.9 mL), compound **2** (100 mg, 0.38 mmol) and 4′-bromopropiophenone (81 mg, 0.38 mmol) in THF (2.5 mL) stirred overnight generated the 4′-bromo-triphenylethylene intermediate (88 mg, 0.20 mmol, 52%). According to general procedure B, 4′-bromo-triphenylethylene intermediate (40 mg, 0.09 mmol) and dimethylamine (2.0 M in THF, 0.9 mL, 1.8 mmol) generated the title compound **271** as an off white solid, *E*/*Z* =1:3.1 (21 mg, 0.046 mmol, 52%). (*Z*)- **271**; ^{1}H NMR (400 MHz, $CDCl_3$) δ 7.36–

7.17 (m, 7H), 7.00–6.94 (m, 2H), 6.75 (d, J = 8.8 Hz, 2H), 6.57 (d, J = 8.8 Hz, 2H), 4.04 (t, J = 5.5 Hz, 2H), 2.84 (t, J = 5.5 Hz, 2H), 2.42 (s, 6H), 2.42–2.37 (m, 2H), 0.89 (t, J = 7.4 Hz, 3H). (*E*)- **271**; ^{1}H NMR (400 MHz, $CDCl_3$) δ 7.25–7.07 (m, 6H), 7.04–6.81 (m, 7H), 4.17 (t, J = 5.5 Hz, 2H), 2.91 (t, J = 5.5 Hz, 2H), 2.47 (s, 6H), 2.45–2.42 (m, 2H), 0.91 (t, J = 7.4 Hz, 3H). HRMS (EI): m/z calculated for $C_{28}H_{33}NO$ $[M+1]^+$: 450.1427; found, 450.1425. The spectroscopic data were consistent with those available in the literature.

N-(2-(4-(4-chloro-1,2-diphenylbut-1-en-1-yl)phenoxy)ethyl)propan-2-amine (343)

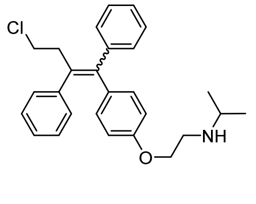

According to general procedure B, compound **2** (100 mg, 0.38 mmol) and isopropylamine (1.6 mL, 19 mmol) in 2-propanol (3.8 mL) generated the isopropylamine-benzophenone intermediate (84 mg, 0.30 mmol, 78%). According to general procedure A, titanium tetrachloride (0.1 mL, 0.9 mmol), Zn dust (118 mg, 1.8 mmol) in THF (1.5 mL), isopropylamine-benzophenone intermediate (84 mg, 0.3 mmol) and 3-chloropropiophenone (51 mg, 0.3 mmol) in THF (2 mL) stirred overnight generated the title compound **343** as an orange oil, *E*/*Z* =1:3.2 (43 mg, 0.10 mmol, 34%). (*Z*)- **343**; ^{1}H NMR (500 MHz, $CDCl_3$) δ 7.41–7.36 (m, 2H), 7.33–7.29 (m, 2H), 7.25–7.13 (m, 6H), 6.80 (d, J = 8.6 Hz, 2H), 6.58 (d, J = 8.7 Hz, 2H), 3.99 (t, J = 5.2 Hz, 2H), 3.43 (t, J = 8.7 Hz, 2H), 2.99–2.92 (m, 5H), 1.12 (d, J = 6.3 Hz, 6H). (*E*)- **343**; ^{1}H NMR (500 MHz, $CDCl_3$) δ 7.17–7.12 (m, 7H), 7.05–7.00 (m, 3H), 6.96–6.88 (m, 4H), 4.15 (t, J = 5.2 Hz, 2H), 3.45 (t, J = 8.7 Hz, 2H), 3.07 (t, J = 5.2 Hz, 2H), 3.02–2.90 (m, 3H), 1.18 (d, J = 6.3 Hz, 6H). HRMS (EI): m/z calculated for $C_{27}H_{30}ClNO$ $[M+1]^+$: 420.2089; found, 420.2085.

Synthesis of Compounds 336–339

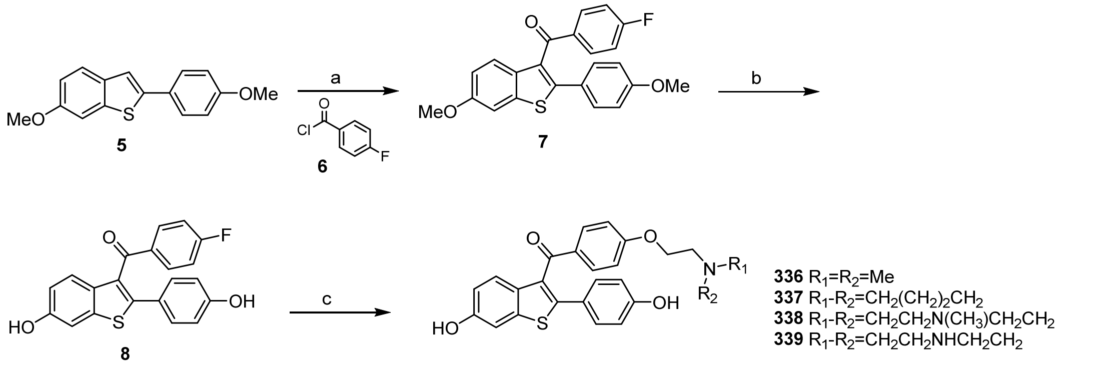

Reagents and conditions: (a) $AlCl_3$, DCM, 0°C-rt, 6h; (b) BBr_3, DCM, 0°C, 3h; (c) alkanolamine, NaH, DMF, 50°C, 16 h

Figure S2. Synthesis of compounds **336–339.**

Experimental Section

Synthesis of (4-fluorophenyl)(6-methoxy-2-(4-methoxyphenyl)benzo[b]thiophen-3-yl)methanone (7)[11]

A solution of 6-methoxy-2-(4-methoxyphenyl)benzo[b]thiophene (**5**, 500mg, 1.8mmol) and 4-fluorobenzoyl chloride (**6**, 0.21 mL, 1.8 mmol) in dry DCM (9 mL) was added dropwise to a solution of $AlCl_3$ (264 mg, 1.98 mmol) in dry DCM (9 mL) at 0 °C under inert atmosphere. The reaction mixture was stirred for 6 h, allowing it to slowly warm to ambient temperature. The reaction mixture was quenched with water (25 mL), extracted with DCM (25 mL x3), the combined organic layers were dried over Na_2SO_4 and concentrated under reduced pressure. The crude was purified by Biotage flash chromatography (gradient elution, 0–10% EtOAc in hexanes) to afford compound **7** as an off-white solid (686 mg, 1.7 mmol, 97%). ^{1}H NMR (400 MHz, $CDCl_3$) δ 7.76 (dd, J = 8.6, 5.6 Hz, 2H), 7.60 (d, J = 8.9 Hz, 1H), 7.33 – 7.24 (m, 3H), 6.98 (dd, J = 8.9, 2.2 Hz, 1H), 6.91 (t, J = 8.6

Hz, 2H), 6.72 (d, J = 8.6 Hz, 2H), 3.86 (s, 3H), 3.73 (s, 3H). The spectroscopic data were consistent with those available in the literature.

Synthesis of (4-fluorophenyl)(6-hydroxy-2-(4-hydroxyphenyl)benzo[b]thiophen-3-yl)methanone (8)[11]

To a solution of compound **7** (680 mg, 1.7 mmol) in dry DCM (5.8 mL) at 0 °C was added BBr_3 (1.0 M in DCM, 5.2 mL, 5.2 mmol) under inert atmosphere. The reaction mixture was stirred for 3 h at 0 °C, poured on ice, quenched slowly with methanol (15 mL), and concentrated under reduced pressure. Then, H_2O (20 mL) was added and extracted with DCM (20 mL x 3), the combined organic fractions were dried over Na_2SO_4. The crude solution was concentrated under reduced pressure and purified by Biotage flash chromatography (gradient elution, 0–30% EtOAc in hexanes) to afford compound **8** as a yellow solid (600 mg, 1.64 mmol, 95%). ^{1}H NMR (400 MHz, CD_3OD) δ 7.70 (dd, J = 8.1, 5.8 Hz, 2H), 7.50 (d, J = 8.8 Hz, 1H), 7.24 (d, J = 2.0 Hz, 1H), 7.11 (d, J = 8.4 Hz, 2H), 6.95 (t, J = 8.6 Hz, 2H), 6.87 (dd, J = 8.8, 2.3 Hz, 1H), 6.59 (d, J = 8.6 Hz, 2H). The spectroscopic data were consistent with those available in the literature.

General Procedure C: Nucleophilic Aromatic Substitution to Synthesize Compounds 336–339

To a solution of the alkanolamine (0.44 mmol) in DMF (1.1 mL) was added NaH (0.48 mmol) and stirred for 30 minutes. Compound **8** (0.11 mmol) was added and the reaction mixture was stirred at 50 °C for 16 h and the solvent was removed under reduced pressure. The crude was purified by Biotage flash chromatography (gradient elution, 0–10% MeOH in DCM) to afford compounds **336–339.**

(4-(2-(dimethylamino)ethoxy)phenyl)(6-hydroxy-2-(4-hydroxyphenyl)benzo[b]thiophen-3-yl)methanone (336)[11]

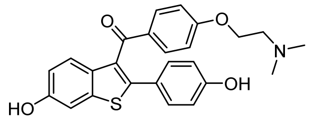

According to general procedure C, compound **8** (40 mg, 0.11mmol), 2-(dimethylamino)-ethanol (0.04 mL, 0.44 mmol), and NaH (19 mg, 0.48 mmol) in DMF (1.1 mL) generated the title compound **336** as a yellow solid (11 mg, 0.025 mmol, 22%). ^{1}H NMR (400 MHz, CD_3OD) δ 7.67 (d, J = 8.9 Hz, 2H), 7.39 (d, J = 8.8 Hz, 1H), 7.24 (d, J = 2.2 Hz, 1H), 7.15 (d, J = 8.6 Hz, 2H), 6.87–6.78 (m, 3H), 6.60 (d, J = 8.6 Hz, 2H), 4.08 (t, J = 5.4 Hz, 2H), 2.77 (t, J = 5.3 Hz, 2H), 2.33 (s, 6H); HRMS (EI): m/z calculated for $C_{25}H_{23}NO_4S$ $[M+1]^+$: 434.1421; found, 434.1416. The spectroscopic data were consistent with those available in the literature.

(6-hydroxy-2-(4-hydroxyphenyl)benzo[b]thiophen-3-yl)(4-(2-(pyrrolidin-1-yl)ethoxy)phenyl)methanone (337)[11]

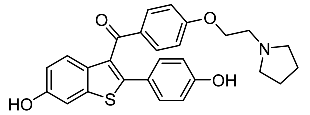

According to general procedure C, compound **8** (40 mg, 0.11mmol), pyrrolidine-1-ethanol (0.05 mL, 0.44 mmol) and NaH (19 mg, 0.48 mmol) in DMF (1.1 mL) generated the title compound **337** as a yellow solid (11 mg, 0.024 mmol, 22%). ^{1}H NMR (400 MHz, CD_3OD) δ 7.68 (d, J = 8.9 Hz, 2H), 7.40 (d, J = 8.8 Hz, 1H), 7.24 (d, J = 2.2 Hz, 1H), 7.15 (d, J = 8.7 Hz, 2H), 6.91–6.76 (m, 3H), 6.59 (d, J = 8.6 Hz, 2H), 4.16 (t, J = 5.3 Hz, 2H), 3.12 (t, J = 5.2 Hz, 2H), 2.98–2.80 (m, 4H), 1.96 – 1.82 (m, 4H); HRMS (EI): m/z calculated for $C_{27}H_{25}NO_4S$ $[M+1]^+$: 460.1577; found, 460.1572. The spectroscopic data were consistent with those available in the literature.

(6-hydroxy-2-(4-hydroxyphenyl)benzo[b]thiophen-3-yl)(4-(2-(4-methylpiperazin-1-yl)ethoxy)phenyl)methanone (338)[11]

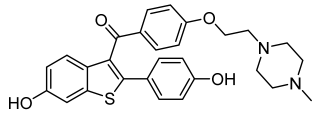

According to general procedure C, compound **8** (40 mg, 0.11mmol), 4-methylpiperazine-1-ethanol (0.06 mL, 0.44 mmol), and NaH (19 mg, 0.48 mmol) in DMF (1.1 mL) generated the title compound **338** as a yellow solid (15 mg, 0.031 mmol, 28%). ^{1}H NMR (400 MHz, CD_3OD) δ 7.66 (d, J = 8.9 Hz, 2H), 7.39 (d, J = 8.8 Hz, 1H), 7.24 (d, J = 2.2 Hz, 1H),

7.15 (d, *J* = 8.6 Hz, 2H), 6.84 (dd, *J* = 8.8, 2.3 Hz, 1H), 6.79 (d, *J* = 8.9 Hz, 2H), 6.59 (d, *J* = 8.6 Hz, 2H), 4.08 (t, *J* = 5.4 Hz, 2H), 2.75 (t, *J* = 5.3 Hz, 2H), 2.67–2.45 (m, 8H), 2.31 (s, 3H); HRMS (EI): m/z calculated for $C_{28}H_{28}N_2O_4S$ $[M+1]^+$: 489.1843; found, 489.1839. The spectroscopic data were consistent with those available in the literature.

(6-hydroxy-2-(4-hydroxyphenyl)benzo[b]thiophen-3-yl)(4-(2-(piperazin-1-yl)ethoxy)phenyl)methanone (339)[11]

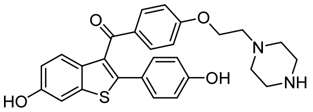

According to general procedure C, compound **8** (40mg, 0.11mmol), 1-Boc-4-(2-hydroxyethyl)piperazine (0.05 mL, 0.44 mmol), and NaH (19 mg, 0.48 mmol) in DMF (1.1 mL) generated Boc-piperazine intermediate which was used for the next step without further purification. The crude was dissolved in DCM/TFA (v/v 1:1, 0.1M), stirred at ambient temperature for 3 h, and concentrated under reduced pressure. The crude was purified by Biotage flash chromatography (gradient elution, 0–50% MeOH in DCM) to afford the title compound **339** as a yellow oil (20 mg, 0.042 mmol, 61%). ^{1}H NMR (400 MHz, CD_3OD) δ 7.68 (d, *J* = 8.9 Hz, 2H), 7.39 (d, *J* = 8.8 Hz, 1H), 7.24 (d, *J* = 2.2 Hz, 1H), 7.15 (d, *J* = 8.6 Hz, 2H), 6.88–6.82 (m, 3H), 6.60 (d, *J* = 8.6 Hz, 2H), 4.24 (t, *J* = 4.9 Hz, 2H), 3.42–3.35 (m, 4H), 3.26–3.18 (m, 6H); HRMS (EI): m/z calculated for $C_{27}H_{26}N_2O_4S$ $[M+1]^+$: 475.1686; found, 475.1684. The spectroscopic data were consistent with those available in the literature.

References

1. Zhang, J., Yan, Z., Wang, S., She, M., Zhang, Z., Cai, W., Liu, P., Li, J. Water soluble chemosensor for Ca^{2+} based on aggregation-induced emission characteristics and its fluorescence imaging in living cells. *Dyes. Pigm.* **150**, 112–120, doi: 10.1016/j.dyepig.2017.11.012.
2. Abdellatif, K.R.A., Belal, A., Omar, H.A. Design, synthesis and biological evaluation of novel triaryl (*Z*)-olefins as tamoxifen analogues. *Bioorg. Med. Chem. Lett.* **23**, 4960–4963, doi: 10.1016/j.bmcl.2013.06.056.
3. Kelly, P.M., Keely, N.O., Bright, S.A., Yassin, B., Ana, G., Fayne, D., Zisterer, D.M., Meegan, M.J. Novel selective estrogen receptor ligand conjugates incorporating Endoxifen-Combretastatin and Cyclofenil-Combretastatin hybrid scaffolds: Synthesis and biochemical evaluation. *Molecules 22*, 1440, doi: 10.3390/molecules22091440.
4. Agouridas, V., Laïos, I., Cleeren, A., Kizilian, E., Magnier, E., Blazejewski, J.-C., Leclercq, G. Loss of antagonistic activity of tamoxifen by replacement of one *N*-methyl of its side chain by fluorinated residues. *Bioorg. Med. Chem.* **14**, 7531–7538, doi: 10.1016/j.bmc.2006.07.012.
5. Robertson, D.W., Katzenellenbogen, J. A., Hayes, J. R., Katzenellenbogen, B. S. Antiestrogen basicity-activity relationships: a comparison of the estrogen receptor binding and antiuterotrophic potencies of several analogues of (*Z*)-l,2-diphenyl-l-[4-[2-(dimethylamino)ethoxy]phenyl]-l-butene (Tamoxifen, Nolvadex) having altered basicity. *J. Med. Chem.* **25**, 167–171, doi: 10.1021/jm00344a015.
6. Garrido, J.M.P.J., Quezada, E., Fajin, J.L.C., Cordeiro, M.N.D.S., Garrido, E.M.P.J.,Borges, F. Electrochemical oxidation of tamoxifen revisited. *Int. J. Electrochem. Sci.* **8**, 5710–5723 (2013).
7. Childers, W., Fan, R., Martinez, R., Colussi, D.J., Melenski, E., Liu, Y., Gordon, J., Abou-Gharbia, M., Jacobson, M.A. Novel compounds that reverse the disease phenotype in Type 2 Gaucher disease patient-derived cells. *Bioorg. Med. Chem. Lett.* **30**, 126806, doi: 10.1016/j.bmcl.2019.126806.
8. Cahiez, G., Moyeux, A., Poizat, M. Stereoselective synthesis of triarylethylenes via copper-palladium catalyzed decarboxylative cross-coupling: synthesis of (*Z*)-tamoxifen. *Chem. Commun. (Camb)***50**, 8982–8984, doi: 10.1039/c4cc03752a (2014).
9. Shiina, I., Sano, Y, Makata, K., Kikuchi, T., Sasaki, A., Ikekita, M., Nagahara, Y., Hasome, Y., Yamori, T., Yamazaki, K. Synthesis and pharmacological evaluation of the novel pseudo-symmetrical tamoxifen derivatives as anti-tumor agents. *Biochem. Pharm.* **75**, 1014–1026, doi: 10.1016/j.bcp.2007.11.005 (2008).
10. Robertson, D.W., Katzenellenbogen, J.A. Synthesis of the (*E*) and (*Z*) isomers of the antiestrogen tamoxifen and its metabolite, hydroxytamoxifen, in tritium-labeled form. *J. Org. Chem.* **47**, 2387–2393, doi: 10.1021/jo00133a030.
11. Ervin, S.M., Hanley, R.P., Lim, L., Walton, W.G., Pearce, K.H., Bhatt, A.P., James, L.I., Redinbo, M.R. Targeting Regorafenib-induced toxicity through inhibition of gut microbial ◎-glucuronidases. *ACS. Chem. Biol.* **14**, 2737–2744, doi:10.1021/acschembio.9b00663.
